# Resolving spatially distinct phytohormone response zones in *Arabidopsis thaliana* roots colonized by *Fusarium oxysporum*

**DOI:** 10.1093/jxb/erae516

**Published:** 2025-01-17

**Authors:** Jacob Calabria, Liu Wang, Madlen I Rast-Somssich, Hsiang-Wen Chen, Michelle Watt, Staffan Persson, Tonni Grube Andersen, Alexander Idnurm, Marc Somssich

**Affiliations:** Plant-Fusarium Interactions Research Team, School of BioSciences, University of Melbourne, Parkville, Australia; Crop Root Physiology Lab, School of BioSciences, University of Melbourne, Parkville, Australia; Plant-Fusarium Interactions Research Team, School of BioSciences, University of Melbourne, Parkville, Australia; Plant Cell Biology Lab, School of BioSciences, University of Melbourne, Parkville, Australia; Crop Root Physiology Lab, School of BioSciences, University of Melbourne, Parkville, Australia; Plant-Fusarium Interactions Research Team, School of BioSciences, University of Melbourne, Parkville, Australia; Plant Cell Biology Lab, School of BioSciences, University of Melbourne, Parkville, Australia; Crop Root Physiology Lab, School of BioSciences, University of Melbourne, Parkville, Australia; Plant Cell Biology Lab, School of BioSciences, University of Melbourne, Parkville, Australia; Max Planck Institute for Plant Breeding Research, Cologne, Germany; Cluster of Excellence on Plant Sciences (CEPLAS), Cologne, Germany; Mycology Laboratory, School of BioSciences, University of Melbourne, Parkville, Australia; Plant-Fusarium Interactions Research Team, School of BioSciences, University of Melbourne, Parkville, Australia; Max Planck Institute for Plant Breeding Research, Cologne, Germany; Cluster of Excellence on Plant Sciences (CEPLAS), Cologne, Germany; University of Ghent, Belgium

**Keywords:** *Arabidopsis thaliana*, ethylene, *Fo*5176, *Fusarium oxysporum*, jasmonic acid, salicylic acid, phytohormones, plant–microbe interactions, plant immunity, spatial immunity

## Abstract

Jasmonic acid (JA), ethylene (ET), and salicylic acid (SA) are the three major phytohormones coordinating plant defense responses, and all three are implicated in the defense against the fungal pathogen *Fusarium oxysporum*. However, their distinct modes of action and possible interactions remain unknown, in part because all spatial information on their activity is lacking. Here, we set out to probe this spatial aspect of plant immunity by using live microscopy with newly developed fluorescence-based transcriptional reporter lines. We have created a GreenGate vector collection of Plant Immune system Promoters (GG-PIPs) that allow us to image local activation of immune pathways with single-cell resolution. Using this system, we demonstrated that SA and JA act spatially separately from each other in distinct sets of root cells neighboring the fungal colonization site, while ET contributed to both sets. SA and ET induced the hypersensitive response as a first line of defense, while JA and ET governed active defense against the pathogen in a separate, second line of defense. Such an approach to resolve plant immune responses on an individual cell level has been lacking, and this work demonstrates that this microscopy-based approach can contribute to understanding plant immune responses in detail.

## Teaser

This work describes the spatial compartmentalization of plant immune responses to pathogenic attack with a focus on the major defense-related phytohormones. Such spatial resolution is mostly overlooked in plant–microbe interactions.

## Introduction

Phytohormones coordinate defense responses to pathogenic microbes (Hou and Tsuda, 2022). A plant senses and recognizes a potential pathogen via its microbe-associated molecular patterns (MAMPs), which are highly conserved structures on the outside of the microbe (examples include flagellin in the bacterial flagellum, or chitin in the fungal cell wall) (Y. Wang *et al.*, 2022). Since the root is continuously surrounded by microbes carrying MAMPs in the soil, recognition of a MAMP alone will not trigger the plant immune system. Instead, it is the co-incidence of MAMPs with cell damage caused by a pathogenic microbe that will launch a full immune response (Zhou *et al.*, 2020; Emonet *et al.*, 2021; Ma *et al.*, 2021). MAMPs and damage-associated molecular patterns (DAMPs) are sensed by the plant outside the cell via plasma membrane-localized PATTERN RECOGNITION RECEPTORS (PRRs), which convey signals via an intracellular kinase domain (Lee *et al.*, 2021). Inside the cell, several defense pathways are then activated, including phytohormone-dependent signaling pathways that depend mainly on jasmonic acid (JA), ethylene (ET), or salicylic acid (SA) (Lee *et al.*, 2021; Hou and Tsuda, 2022). Among them, ET mostly acts in concert with JA and SA, while JA and SA are, in all explored cases, mutually antagonistic. In current models, JA controls plant defense against necrotrophic pathogens, while SA is the regulator of pathways combating biotrophic pathogens (Hou and Tsuda, 2022). SA-mediated immunity can involve the hypersensitive response (HR), a form of programmed cell death, to kill the tissue surrounding the infection site, in an attempt to prevent further spread of the invading microbe (Pitsili *et al.*, 2020). This form of defense is not activated against necrotrophic pathogens, as these feed on dead plant material (McCombe *et al.*, 2022). Thus, the JA/ET-mediated response to necrotrophs instead relies on measures such as the release of reactive oxygen species (ROS) into the apoplast, producing antimicrobial metabolites, or the manipulation of the apoplastic pH and environment (Masachis *et al.*, 2016; McCombe *et al.*, 2022). While we have a detailed understanding of the molecular mechanisms underlying these aspects of plant immunity, we lack spatial information on how these responses are coordinated at the single-cell level as well as over time in roots.

*Fusarium oxysporum*, a soil-inhabiting ascomycete fungus, is a destructive pathogen to many plant species, including major crop plants, such as tomato (*Solanum lycopersicum*), cotton (*Gossypium barbadense*), and banana (*Musa acuminata*). *Fusarium oxysporum* hyphae grow in the soil and are chemotactically guided toward the root of their host plant (Turrà *et al.*, 2015; L. Wang *et al.*, 2022). Upon reaching the plant, they attach and grow along the root until they enter via natural openings, such as lateral root emergence sites or wounds (L. Wang *et al.*, 2022). Once inside the vasculature, the fungus drains the plant of water and nutrients, and can spread to all parts of the plant, which begins to wilt and eventually dies (Gordon, 2017). Based on these observations, *F. oxysporum* is considered a hemibiotrophic fungus, that initially lives biotrophically within the vasculature of the host before eventually turning necrotrophic and killing the plant (Gordon, 2017). Early work on the *Arabidopsis thaliana*–*F. oxysporum* strain *Fo*5176 pathosystem has focused on creating several large-scale transcriptomic datasets, as well as comprehensive infection studies of *A. thaliana* mutants, to investigate the role of JA and SA in combating infection by *F. oxysporum* (L. Wang *et al.*, 2022). These studies have established a role for the different hormones in plant defense against *F. oxysporum*, but were also often inconclusive as to their exact roles (L. Wang *et al.*, 2022). Thus, while it is assumed that both SA and JA are involved in plant defense response against this hemibiotrophic fungus, a detailed understanding of their action is lacking (L. Wang *et al.*, 2022).

To address these unknown aspects of plant immunity, we set up a microscopy-based live-imaging approach capable of monitoring transcriptional activity of different pathways *in planta*, with high spatiotemporal resolution. To do this, we produced the GreenGate-based Plant Immune system Promoter (pGG-PIP) vector collection with regulatory sequences for 75 plant immunity genes, representing several pathways of the plant immune system, including the different phytohormones (Calabria *et al.*, 2022). We used these vectors to produce fluorescent *A. thaliana* reporter lines. In combination, these resources can be used to monitor locally and temporally restricted transcriptional activation of the various immune pathways. We detected distinct responses of genes involved in SA, JA, and ET biosynthesis, signaling, and regulation, targeted to a limited number of root cells upon infection with *F. oxysporum*. Combined, our work highlights spatially distinct immune response zones in an infected root. This work thereby establishes a platform for further investigation of plant–microbe root interactions with single-cell resolution.

## Materials and methods

### Cloning of the pGG-PIP transcriptional reporter constructs

The pGG-PIP entry vectors are based on the pGGA000 vector from the original GreenGate kit (Lampropoulos *et al.*, 2013). The different promoters were amplified from *A. thaliana* (natural accession Columbia) DNA, extracted from rosette leaves with the Qiagen DNeasy Plant Kit, and using the primers given in Supplementary Table S1. For error-free amplification, the Phusion high-fidelity DNA polymerase with proofreading (New England Biolabs) was used. The fragments were then transferred into pGGA000 in a Golden Gate assembly reaction using the NEBridge Golden Gate Assembly Kit (BsaI-HF v2) (New England Biolabs). To produce the transcriptional reporter lines, we used the destination vector pGGZ003, as well as the donor vectors pGGD007 (*linker-NLS*), pGGE009 (*UBQ10* terminator), and pGGF005 (*pUBQ10::HygR:tOCS*) from the original GreenGate kit from Lampropoulos *et al.* (2013). Further, the donor vectors pGGB-mT2 (*mTurquoise2*) and pGGC-mT2 (*mTurquoise2*) were created by cloning the *mT2* coding sequence from an AddGene-derived template into pGGB000 and pGGC000 from the original GreenGate kit (Goedhart *et al.*, 2012; Lampropoulos *et al.*, 2013). In combination with our pGG-PIP donor vectors, this yielded the *pPIP::mT2-mT2-NLS:tUBQ10:pUBQ10::HygR:tOCS* reporter contructs.

### Plant growth and transformation

*Arabidopsis thaliana* natural accession Columbia-0 (Somssich, 2018) plants were grown until the flowering stage under day/night conditions of 21 °C/18 °C, 16 h/8 h, with a light intensity of 120 mmol m^–2^ s^–1^ and constant relative humidity of 70%. Plants were transformed using a slightly modified *Agrobacterium tumefaciens*-mediated floral dip protocol (Clough and Bent, 1998; Somssich, 2019). *Agrobacterium tumefaciens* strain GV3101 *pMP90 pSoup* carrying the plasmids of interest were grown in 250 ml of 2× YT medium supplemented with 50 µg ml^–1^ rifampicin, 25 µg ml^–1^ gentamycin, and 100 µg ml^–1^ spectinomycin for 16 h at 28 °C, shaking at 200 rpm (Miller, 1972; Holsters *et al.*, 1980; Koncz and Schell, 1986; Hellens *et al.*, 2000). The *A. tumefaciens* cultures were centrifuged at 3200 *g* for 20 min, and the pellet was resuspended in 300 ml of 5% sucrose solution with 0.08% Silwet L-77 (Clough and Bent, 1998). Plants were inverted and the flowers submerged in *A. tumefaciens* solution for 30 s with agitation. Plants were briefly patted dry to remove excess *A. tumefaciens* before laying them horizontally in a plant tray covered with plastic film to maintain humidity at room temperature overnight. The following day, plants were reverted upright and returned to the original growth conditions. The floral dipping procedure was repeated after a 7 d interval and, upon maturation of siliques, seeds were harvested. Harvested seeds were dried for at least 2 weeks and then surface-sterilized by washing for 2–3 h in 75% ethanol with 0.1% Triton X-100 on an upright tube rotator. The seeds were decanted onto filter paper in a laminar flow cabinet to evaporate the ethanol. The dry seeds were then sprinkled onto a Petri dish with half-strength basal Murashige and Skoog (MS) medium with vitamins supplemented with 30 µg ml^–1^ hygromycin (Murashige and Skoog, 1962). Following a 3–5 d stratification period in darkness at 4 °C, the plates were placed in a growth cabinet for 10–14 d. After this, seedlings that survived selection were transferred to fresh plates without antibiotics and grown until they were too big for the plates, at which point they were transferred to soil until maturation of siliques.

### Fungal growth and transformation

The *F. oxysporum* f. sp. *conglutinans* strain 5176 (*Fo*5176) was used (Y. Wang *et al.*, 2022). To generate a *Fo*5176 line expressing cytoplasmic tdTomato (tdT), an expression clone was generated by cloning the *tdT* coding sequence [amplified from an AddGene-derived plasmid (Shaner *et al.*, 2004)] into the *Bgl*II cloning site of plasmid pLAU2 using Gibson assembly (Idnurm *et al.*, 2017). This placed the gene under the control of a constitutive promoter from the *actin* gene of the ascomycete *Leptosphaeria maculans*. After replication in *Escherichia coli*, this plasmid pMAI32 was electroporated into *A. tumefaciens* strain EHA105 with selection on LB medium with kanamycin (50 µg ml^–1^). *Fo*5176 was routinely cultured on potato dextrose agar (PDA) plates. To generate spores for transformation, five plugs of ~5 mm diameter were inoculated into 50 ml of half-strength potato dextrose broth and cultured at 150 rpm for 5 d at room temperature, then filtered through miracloth, centrifuged at 3000 *g* for 5 min, and resuspended in sterile H_2_O. The spores were then transformed with the *A. tumefaciens* strain that had been cultured overnight in LB broth with kanamycin, using standard methods (Idnurm *et al.*, 2017): fungal spores and bacteria were mixed on induction medium plates and co-cultured for 3 d. Selection for transformants used an overlay of PDA supplemented with hygromycin and cefotaxime (50 µg ml^–1^ and 100 µg ml^–1^, respectively). Transformants that grew through the overlaid medium were subcultured onto PDA containing hygromycin and cefotaxime, and allowed to produce conidia, which were separated with a metal loop to generate strains derived from a single conidium. The strains were further checked for integration of the transgene by PCR, and checking for fluorescence coming from the integrated tdTomato fluorophore after multiple rounds of culturing in the absence of hygromycin, which was used for selection of the transformants.

### Plant–fungus co-cultivation and infection

To obtain fungal spores, *Fo*5176 was grown for at least 7 d on PDA plates at room temperature, at which time five pieces of ~1 mm^2^ were cut from the plate and dropped into a flask containing 50 ml of yeast nitrogen base (YNB) medium with 1% sucrose. The liquid cultures were incubated for 4 d at room temperature with shaking at 120 rpm. The solution was then filtered through miracloth, and the spores were harvested by centrifugation at 3000 *g* for 10 min, and resuspended in 25 ml of sterile ultrapure water. For the co-cultivation of plant and fungus, *A. thaliana* seedlings were grown on a vertical Petri dish containing half-strength basal MS medium with vitamins for 11 d and then transferred to a horizontal Petri dish with a 2–3 cm strip of half-strength basal MS medium with vitamins at the top end, while the rest of the plate was filled ~2–3 mm high with liquid quarter-strength basal MS medium with vitamins (this is a set-up with slight modifications as previously described in Tintor *et al.*, 2020). The seedlings were placed onto the thin MS medium strip at the top end, with the root in the liquid medium. Fungal spores were then added to the liquid medium. The plates were covered with aluminum foil, leaving only the leaves exposed to light, and then placed into a growth chamber.

### Microscopy

We imaged the infection and the progression of colonization on a Leica M205 FA stereomicroscope. Infection could usually be observed on day 3 after spore addition, and at day 5 there was robust colonization. We usually imaged daily from day 3 to 11 d post-infection. The images shown in the figures are from day 4 post-spore addition, when robust colonization was observed in all lines. For the fluorescence coming from the plant 2xmT2 reporters, we used the Leica ET CFP (ET436/×20 ET480/40m) filter, and for the fungal tdT reporter the Leica ET mCHER (ET560/×40 ET630/75m) filter. We use ×80 magnification for the images. The settings for the imaging (illumination strength, exposure time, gain, etc.) were kept constant for all imaging sessions, to allow for semi-quantitative imaging, and to make the images at least relatively comparable. The images were recorded using the Leica Application Suite software, and processed using Fiji Is Just ImageJ (FIJI) and the GNU Image Manipulation Program (GIMP) (Schindelin *et al.*, 2012).

## Results

### The pGG-PIP entry vector collection

In order to study plant immune responses to colonization on an individual cell level, we created a collection of fluorescence-based transcriptional reporter lines by cloning the putative regulatory sequences (i.e. promoters) of 75 *A. thaliana* genes representing many of the major branches of the plant immune system (Supplementary Table S2). These plant immune system promoters were used to control the expression of two fused nuclear-localized mTurquoise2 (mT2-mT2-NLS) fluorescent proteins. These fluorescent reporters are strong and stable in plant cells, and the nuclear localization enhances the signal due to the molecular crowding effect. Nuclear targeting furthermore allows the identification of single cells due to their individual nuclei. This was done using the GreenGate cloning system (Lampropoulos *et al.*, 2013), therefore creating an entry vector set that is compatible with all other GreenGate-based cloning toolkits. Several such kits have been produced since the introduction of GreenGate in 2013, including two updates to expand the functionality of the GreenGate system (Piepers *et al.*, 2023; Pennetti *et al.*, 2024), and toolkits providing entry vector sets to build plasmids for CRISPR/Cas9 [clustered regularly interspaced palindromic repeats (CRISPR)/CRISPR-associated protein 9]-guided genome editing (Wu *et al.*, 2018), CRISPR/Cas9-guided tissue-specific gene knockout (Decaestecker *et al.*, 2019), or inducible and cell type-specific gene expression (Schürholz *et al.*, 2018). Additionally, there are fluorescent reporters available that have been thoroughly characterized *in planta* (Denay *et al.*, 2019).

**Fig. 1.**
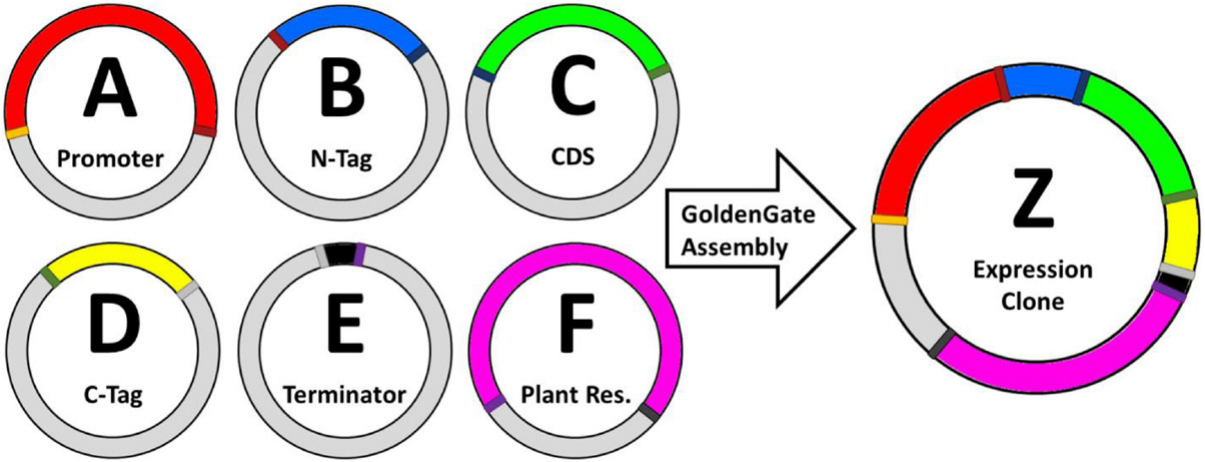
The basic GreenGate expression clone assembly principle. The different genetic modules are cloned into specific entry/donor vectors (here, A–F) defined by their 4 bp overhangs. Gene promoters (red) are typically cloned into the A-module vector and are defined by the orange and brown 4 bp overhangs in this illustration. An N-terminal tag would be cloned into the B-module vector, and is defined by the brown and navy blue overhangs. In the following assembly reaction, the promoter module and the N-tag module would self-assemble due to the matching orange 4 bp overhangs. Similarly, the other fragments donated by the C, D, E, and F module vectors also self-assemble into one long DNA fragment, which then self-assembles via the two terminal overhangs (orange from A and dark green from F, in this illustration) into the Z-module destination/expression vector. Thus, a single assembly reaction produces one ordered expression clone with fragments from six entry/donor vectors. Depending on the GreenGate toolkit used, more modules can be used to produce higher order vectors.

The GreenGate cloning system is a modular cloning system based on Golden Gate assembly, and uses recombination-based hierarchical assembly of multiple donor modules (each containing, for example, a promoter of choice, an N- and/or C-terminal tag of choice, the gene of interest, a reporter gene of choice, a resistance cassette, etc.) into one ordered expression clone to be used for transgenesis in a single fast reaction (Fig. 1). The ordered assembly of the various donor fragments from the entry clones into one expression clone is based on the use of defined 4 bp overhangs for each module, and thus all published kits and vectors can be used in mix-and-match approaches, as long as these defined overhangs are used (Fig. 1). The 75 pGG-PIP vectors in our collection are therefore compatible with all other toolkits to be used as promoter entry vectors, and a valuable resource for the plant science community. We donated the 75 entry vectors to AddGene for distribution.

To monitor the infection and colonization of *A. thaliana* roots by *F. oxysporum*, we cultivated 10-day-old seedlings in square Petri dishes, added fungal spores, and then imaged hyphal growth and colonization of the plant root. To visualize hyphae, we used a transgenic *F. oxysporum* line that expresses a cytoplasmic tdTomato fluorophore.

### *F. oxysporum* colonization of the root leads to cell death of the infected tissue

**Fig. 2.**
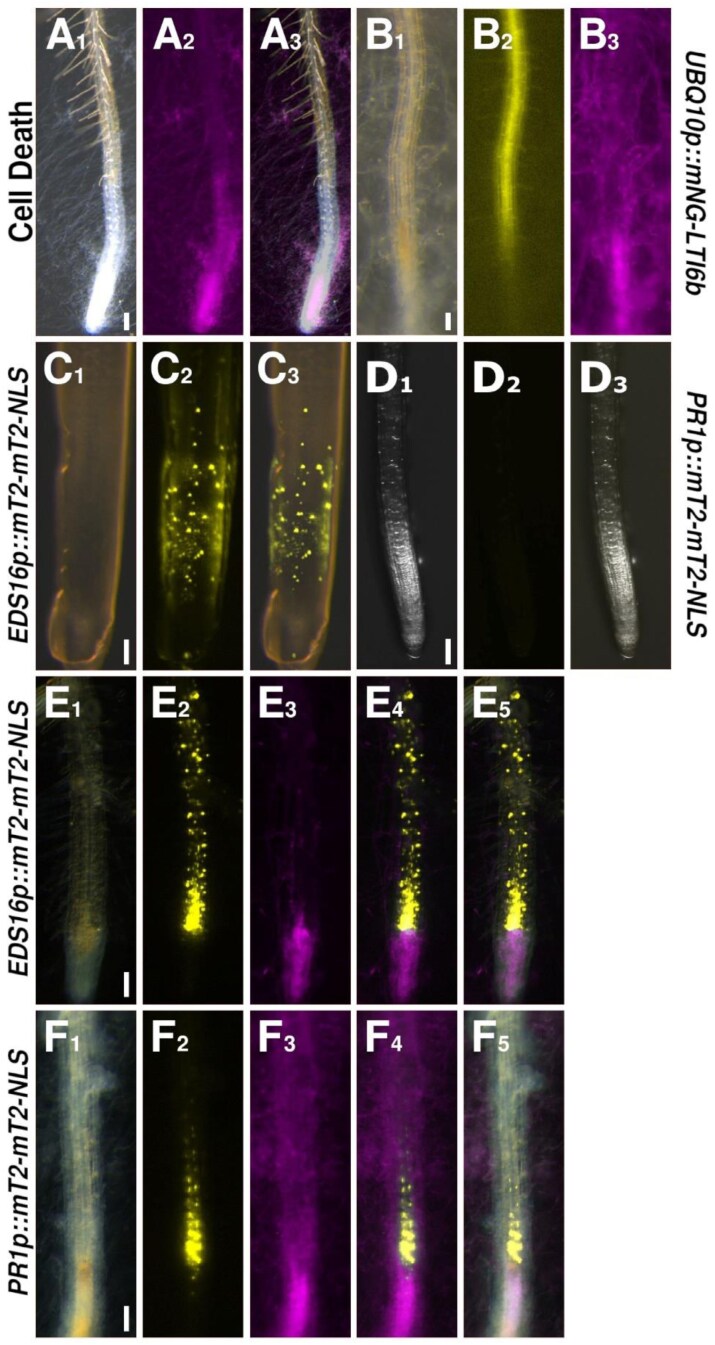
Cell death response of colonized roots and expression patterns of salicylic acid (SA)-related reporters. (A) A root tip colonized by *F. oxysporum* (magenta). (B) A colonized root tip expressing the plasma membrane marker LTI6b (yellow). (C, E) The SA biosynthesis reporter *EDS16* (yellow) under control (C) or colonized (E) conditions. (D, F) The SA signaling reporter *PR1* (yellow) under control (D) or colonized (F) conditions. Scale bars are 100 µm.

One of the first things we noticed when imaging the colonization process was that *F. oxysporum* always colonizes the plant via the root tip. Furthermore, colonization of a root tip appeared to cause immediate localized cell death of the entire tip (Fig. 2A). Dead root tips leave behind cell wall material, which could be observed as a bright white signal in the image (Fig. 2A). By imaging plants expressing a fluorescent marker targeted to the plant plasma membrane [mNeonGreen(mNG)-LTI6b], this became even more apparent, as the fluorescence signal from the plasma membrane outlined all cells of the plant but disappeared completely from cells around the colonized tissue (Fig. 2B), which supports that these cells underwent cell death as a response to the fungus. Intriguingly, while fungal colonization progressed through the vasculature, cell death spanned the entire circumference of the root, including the outer tissues, which are not colonized by the fungus. Because cell death was not limited to the colonized tissue, we reasoned that cell death may be the result of an HR initiated by the plant, rather than caused directly by the fungus.

### Salicylic acid biosynthesis is up-regulated in the vascular cells in direct contact with the colonized tissue

To investigate if the observed cell death of the infected root tips is indeed caused by an HR of the plant, we employed the subset of pGG-PIP reporters associated with the HR. The HR is typically the result of high local SA and ET concentrations, as well as the sensing of cell damage, and an associated ROS burst (Pitsili *et al.*, 2020). As proxies for these events, we chose the pGG-PIP reporters for *ENHANCED DISEASE SUSCEPTIBILITY 16* [*EDS16*; also named *SALICYLIC ACID DEFICIENT 2* (*SID2*)], a key SA biosynthetic enzyme (vector pGG-PIP53), *PATHOGENESIS-RELATED PROTEIN 1* (*PR1*), an SA signaling-responsive and hypersensitive response marker (pGG-PIP56), and *1-AMINOCYCLOPROPANE-1-CARBOXYLIC ACID SYNTHASE 2* (*ACS2*), a key enzyme in the ET biosynthesis pathway (pGG-PIP50). Further, we used markers for *PLANT ELICITOR PEPTIDE 1* (*PEP1*), mobile peptides that are released when a cell is damaged and thus act as DAMP signaling molecules (pGG-PIP01), as well as *PEP RECEPTOR 1* and *2* (*PEPR1* and *PEPR2*), the receptors for PEP1 (pGG-PIP16 and pGG-PIP17). Finally, *BOTRYTIS-INDUCED KINASE1* (*BIK1*) (pGG-PIP22), a cytoplasmic kinase that is activated via phosphorylation from PRRs such as the PEPRs, and then in turn activates the NADPH-oxidase RESPIRATORY BURST OXIDASE HOMOLOG D (RBOHD) (pGG-PIP31) to produce a ROS burst, was used (Supplementary Table S2).

In uninfected control roots, the *EDS16-*based reporter showed activity in the meristematic and elongation zones (MZ/EZ) of the root tip, as well as in older differentiated cells, with no signal detectable in young differentiating cells (Fig. 2C). In the tip, expression appeared to be stronger in the outer layers of the EZ (Fig. 2C). *PR1* activity was only weakly detectable in the mature differentiation zone, but was absent from the root tip in our controls (Fig. 2D). However, following infection and colonization by *F. oxysporum*, *EDS16* expression appeared to be up-regulated in the root tip, and spatially targeted to a small group of vascular cells immediately adjacent to the tissue already colonized by the fungus (Fig. 2E). *PR1* reporter expression, which was not detectable in the tip of the uninfected control, was also up-regulated in these same cells (Fig. 2F). Notably, weaker *EDS16* expression could also be observed stretching up the root, while *PR1* was limited to the set of cells likely to subsequently initiate an HR (Fig. 2F). Importantly, this up-regulation appeared to be confined to cells of the vasculature, the tissue targeted by *F. oxysporum*.

### Local up-regulation of ET biosynthesis and signaling only partially overlaps the SA zone

**Fig. 3.**
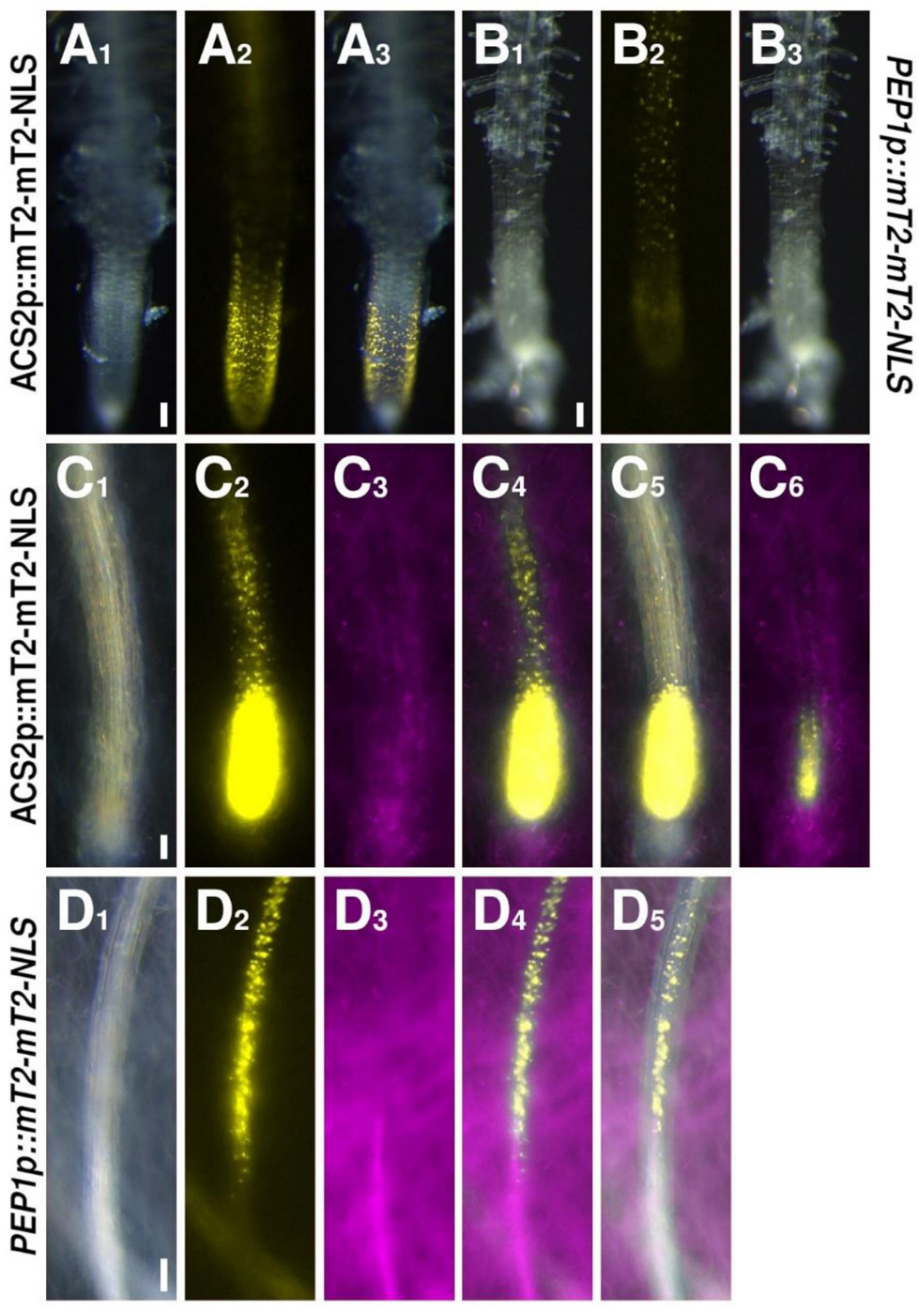
Expression patterns of ethylene (ET) biosynthesis and *PEP1* reporters. (A, C) The ET biosynthesis reporter *ACS2* (yellow) under control (A) or *F. oxysporum*- (magenta) colonized (C) conditions. The image in C_6_ is the same root as in C_1–5_, but was recorded with reduced exposure time to allow for the identification of individual cells. (B, D) The *PEP1* reporter (yellow) under control (B) or *F. oxysporum*- (magenta) colonized (D) conditions. Scale bars are 100 µm.

We then investigated the responsiveness of our ET biosynthesis reporter. The *ACS2* reporter was expressed throughout many tissues of the plant, likely to be a representation of this hormone acting not only in immunity, but also in several developmental pathways which are also active under our control conditions. In the root tip, there was expression in the meristem, which became weaker in the EZ (Fig. 3A). Toward the end of the EZ and with the beginning of the root hair zone, expression was absent (Fig. 3A). In response the fungal colonization, however, the *ACS2* reporter showed strong up-regulation in a group of cells next to the colonized tissue (Fig. 3C). In fact, the expression was so strong, that we could not resolve individual cells under our standard imaging conditions used for all images in the study (Fig. 3C_2,4,5_), and we thus had to reduce the exposure time to visualize the nuclei (Fig. 3C_6_). The maximum *ACS2* reporter expression was in the cells immediately adjacent to the colonized tissue, and thus overlapping the maximum *EDS16* expression. However, signal from the *ACS2* reporter appeared to stretch up the root, and thus seemed broader than the *EDS16* reporter pattern. Based on these observations, there seems to indeed be an overlap between the expression maxima of SA and ET biosynthesis in the cells that we hypothesize will next induce the HR.

### Damage-associated signaling is activated in the cells bordering the infection site

**Fig. 4.**
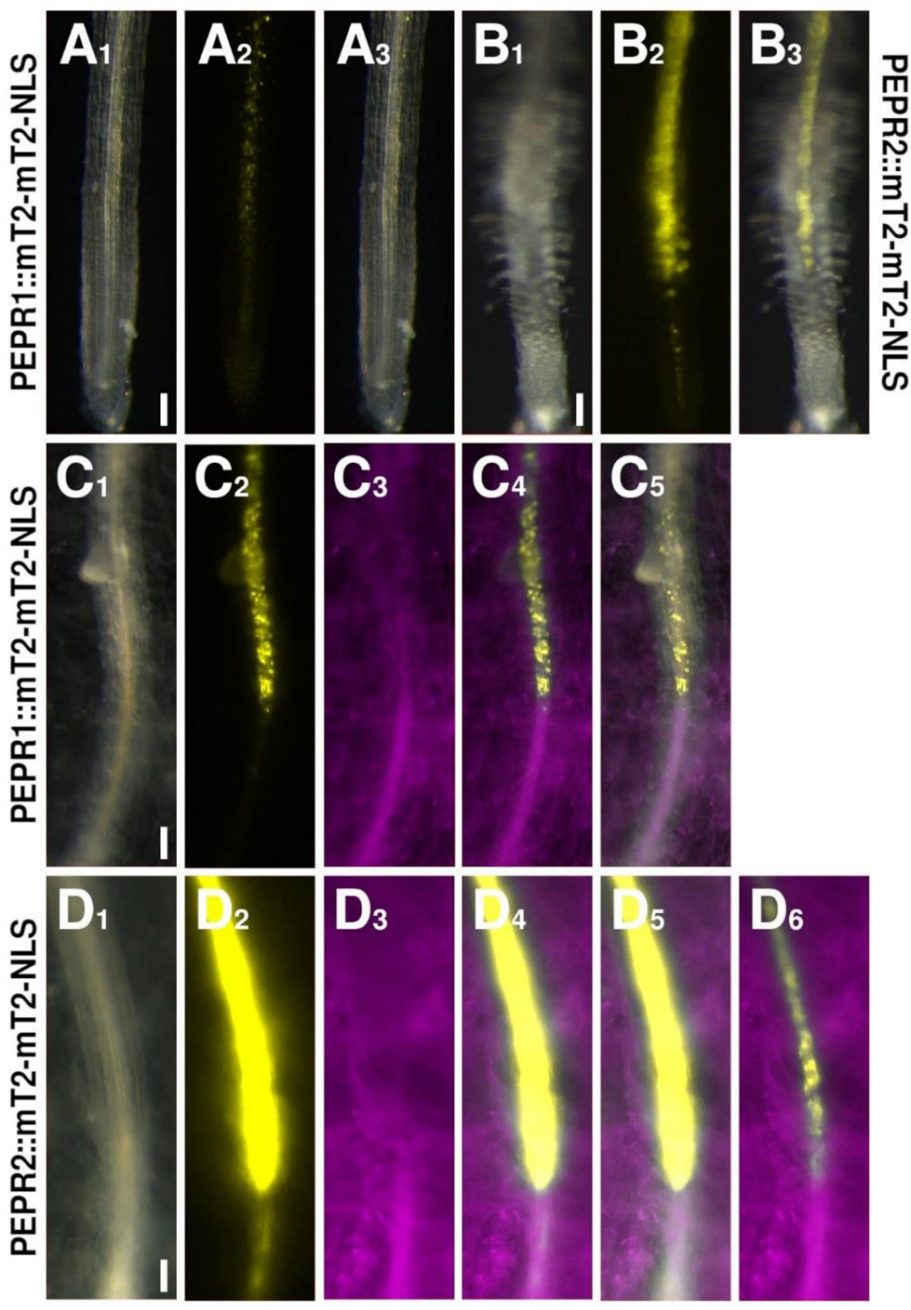
Expression patterns of the *PEPR* reporters. (A, C) The *PEPR1* reporter (yellow) under control (A) or *F. oxysporum*- (magenta) colonized (C) conditions. (B, D) The *PEPR2* reporter (yellow) under control (B) or *F. oxysporum*- (magenta) colonized (D) conditions. The image in D_6_ is the same root as in D_1–5_, but was recorded with reduced exposure time to allow for the identification of individual cells. Scale bars are 100 µm.

Next to high levels of SA and ET, another requirement for the induction of the HR is the sensing of cell damage, as the plant will only induce a response as drastic as programmed cell death if the pathogen is actually causing damage. To assess the influence of DAMP responses, we employed our PEP reporters. The PEP pathway functions as a damage sensor in response to several plant pathogens, including bacteria, fungi, and oomycetes (Saijo *et al.*, 2018). The PEP peptides function as phytosulfokines. They are expressed as part of the plant pattern-triggered immunity (PTI) response, and trigger cell-autonomous and non-autonomous defense responses when they are perceived by the plasma membrane-localized receptors PEPR1 and PEPR2 (Yamaguchi *et al.*, 2006, 2010; Saijo *et al.*, 2018). Importantly, the peptides do not have a signal peptide to target them to the apoplast. Hence, they are only released by a cell if the plasma membrane is damaged to allow for their release. Therefore, they can act as DAMP messengers. Perception of the PEPs by the PEPRs then triggers downstream immune responses, including a ROS burst (Liu *et al.*, 2013; Kadota *et al.*, 2014; Saijo *et al.*, 2018; Jing *et al.*, 2020). Under control conditions, *PEP1* was expressed in inner tissues of the root differentiation zone, with only faint expression detectable in the EZ of the root tip (Fig. 3B). The two *PEPR* genes were generally expressed in the root vasculature, starting either in or just after the EZ (Fig. 4A, B). *PEPR2* expression was stronger compared with *PEPR1* (Fig. 4A, B). Following colonization of the vasculature by *F. oxysporum*, *PEP1*, as well as both *PEPR* reporters, showed a strong up-regulation in the vascular cells starting at the colonization site, but stretching much further up the root than the more local responses observed for SA and ET biosynthesis (Figs 3D, 4C, D). Importantly, the induction of *PEPR2* was much stronger than the induction of *PEP1* and *PEPR1*; in fact, as for the ET biosynthesis reporter *ACS2*, we had to reduce the exposure time for PEPR2, to visualize individual cells (Fig. 4D_6_). The observation that this pathway is not just locally up-regulated, but stretches much further up the root, could be a reflection of this pathway being involved in cell-to-cell signaling, to relay the information of pathogen infection to other tissues.

### ROS reporters overlap with the SA, ET, and DAMP reporters in the HR zone

**Fig. 5.**
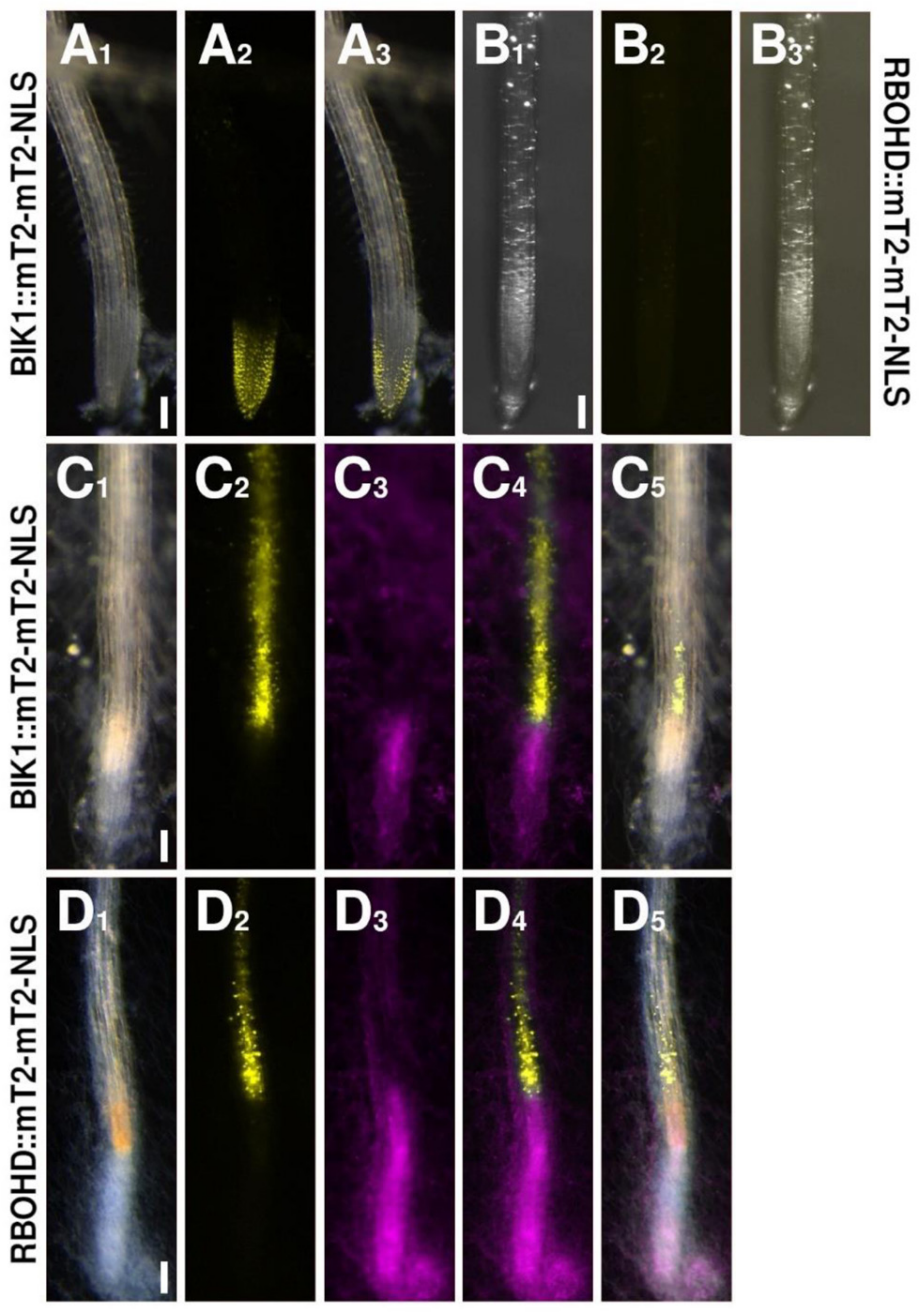
Expression patterns of the *BIK1* and *RBOHD* reporters. (A, C) The *BIK1* reporter (yellow) under control (A) or *F. oxysporum*- (magenta) colonized (C) conditions. (B, D) The *RBOHD* reporter (yellow) under control (B) or *F. oxysporum*- (magenta) colonized (D) conditions. Scale bars are 100 µm.

Finally, to complete our set of HR markers, we turned to BIK1 and RBOHD. The kinase gene *BIK1* is expressed in the MZ of the root tip, with expression appearing stronger in the outer tissue, the columella, but being absent from the EZ (Fig. 5A). Following colonization of the root by *F. oxysporum*, this pattern changed significantly, with the *BIK1* reporter being induced in the inner tissues of the root tip, with a visible maximum of expression in the vascular cells immediately neighboring the colonized tissue (Fig. 5C). Activation of BIK1 via PRRs reportedly results in phosphorylation and activation of the oxidase RBOHD to produce a local ROS burst (Kadota *et al.*, 2014). Under uninfected control conditions, the *RBOHD* reporter is expressed in all cells and tissues of the differentiated root, but not, or only faintly, in the root tip (Fig. 5B). Upon infection by *F. oxysporum*, expression is activated in the cells next to the colonization site, mirroring the pattern we observed for *BIK1* (Fig. 5D). We also regularly observed ‘root browning’ of the cells at the colonization site, which may be the result of the local ROS burst produced by RBOHD (visible, for instance, in Fig. 5D_1_).

Hence, the expression maxima of the SA and ET biosynthesis and signaling, DAMP signaling, and ROS reporters all overlapped in the small group of vascular cells closest to the colonized tissue (Figs 2–5). Combining these findings with our observation that an infected root tip immediately dies off suggests that an HR is indeed induced and responsible for the observed cell death. Furthermore, we found that the PEP and ROS signals extend further up the root than the very local hormones responses, indicating a role for these mobile molecules in priming cells further up the root for impeding attack by the pathogen.

### Jasmonic acid and WRKY11 define a second, distinct response zone next to the HR zone

**Fig. 6.**
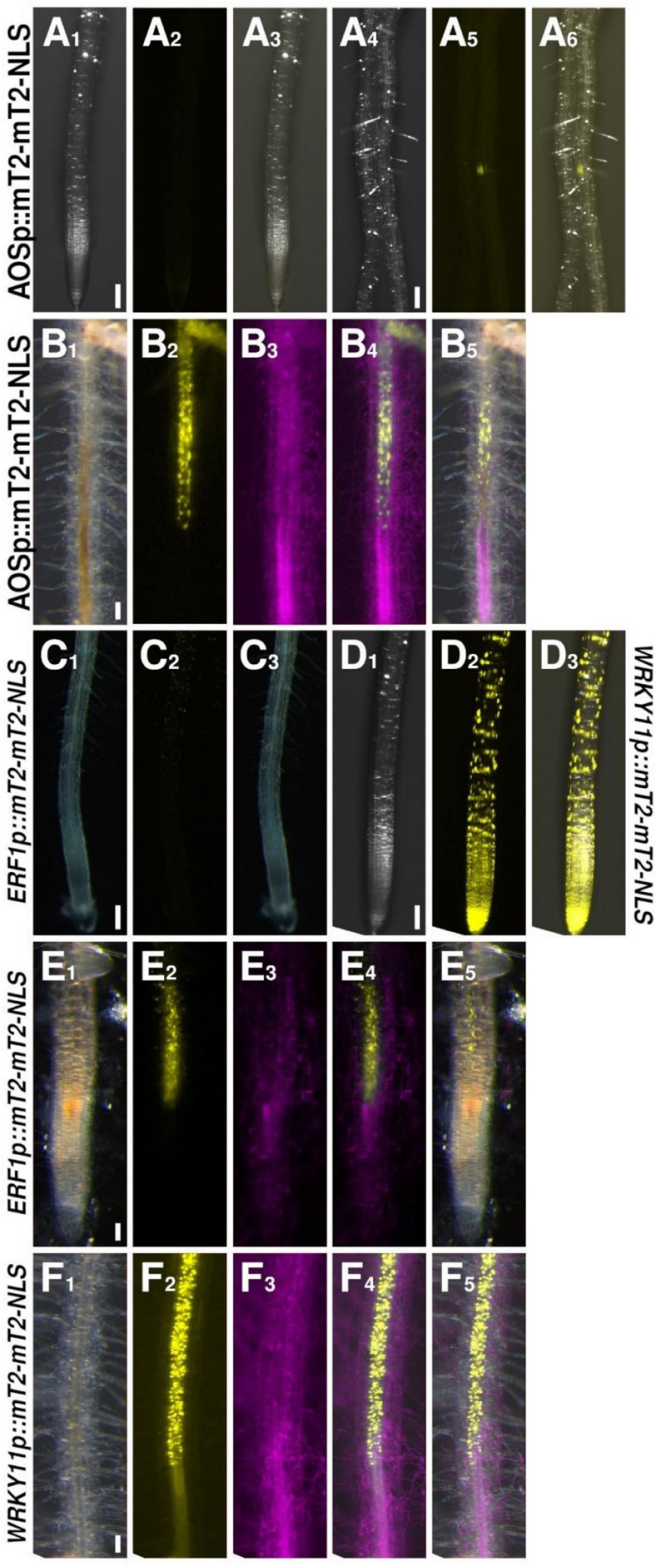
Expression patterns of the *AOS*, *ERF1*, and *WRKY11* reporters. (A, B) The *AOS* reporter (yellow) under control (A) or *F. oxysporum*- (magenta) colonized (B) conditions. (C, E) The *ERF1* reporter (yellow) under control (C) or *F. oxysporum*- (magenta) colonized (E) conditions. (D, F) The *WRKY11* reporter (yellow) under control (D) or *F. oxysporum*- (magenta) colonized (F) conditions. Scale bars are 100 µm.

JA is the third major stress hormone in plants next to SA and ET, and we thus investigated its role in the *F. oxysporum* interaction. *ALLENE OXIDE SYNTHASE* (*AOS*) (pGG-PIP51) is a key enzyme in the JA biosynthesis pathway. *AOS* reporter activity was mostly absent in the uninfected control roots, with very distinct points of expression limited to lateral root emergence sites (Fig. 6A). Following infection and colonization of the plant by *F. oxysporum*, *AOS* expression was robustly induced in a distinct group of cells (Fig. 6B). Interestingly, this group of cells did not immediately border the colonized cells, as the cells that expressed the SA reporters did. Instead, they seemed to be several cells removed from the colonized tissue. The expression then slowly tapered along the root toward the base. Again, expression was limited to cells of the vasculature, with no up-regulation in the outer tissues (Fig. 6B). Thus, JA biosynthesis, like SA biosynthesis, is likely to be targeted to the fungal colonization site; however, with these two phytohormones being mutually antagonistic, both appear to specify their own, individual and spatially separate response zones in the infected tip. To investigate the activation of JA signaling, we then imaged the reporter for *ETHYLENE RESPONSE FACTOR 1* (*ERF1*) (pGG-PIP54). ERF1 is an activator of JA- and ET-responsive defense gene expression, as well as an integrator of the JA and ET signaling pathways (L. Wang *et al.*, 2022). In uninfected roots, signal from the *ERF1* reporter was visible in differentiated tissues, but was absent from the root tip, including the MZ and EZ (Fig. 6C). Upon infection, *ERF1* was up-regulated in a pattern that resembled the one from *AOS* (Fig. 6E).

**Fig. 7.**
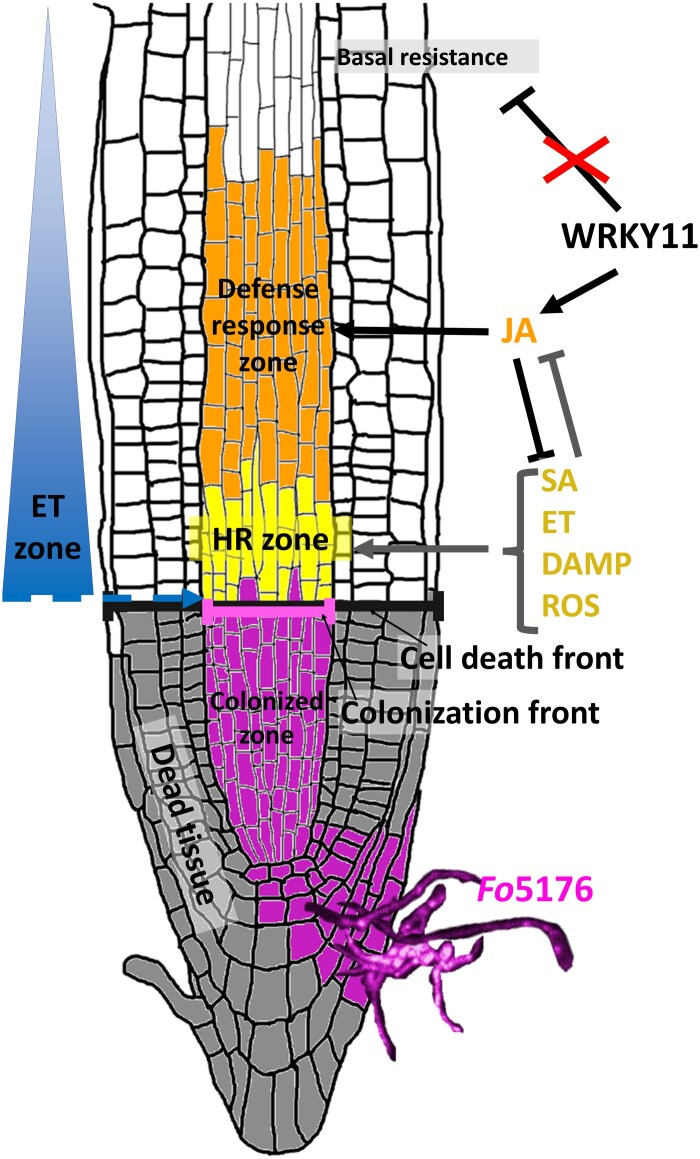
Model showing the two distinct response zones. *F*. *oxysporum* infects an *A. thaliana* root tip via the meristematic zone, where it colonizes the vasculature (purple cells=colonized zone). The tissue around this zone undergoes immediate programmed cell death (dark gray cells). As the colonization front (light purple line) progresses in the vasculature, so does a cell death front (black line) across all tissues. In an attempt to prevent the fungus from infecting more tissue and spreading through the vasculature to all parts of the plant, the plant triggers the HR in a small, spatially confined group of cells immediately adjacent to the colonization front (yellow cells), the HR zone. HR is activated by SA in combination with ET, ROS, and DAMP signaling. Slightly removed from the HR and colonized zones, the plant launches a second line of defense to combat the spread of the pathogen in the defense response zone (orange cells). This response is dependent on WRKY11 and JA/ET biosynthesis and signaling. JA and SA are mutually antagonistic, thereby establishing the two spatially separate zones of distinct action. WRKY11 is furthermore a negative regulator of basal resistance. Upon colonization by *F. oxysporum*, the plant therefore represses *WRKY11* in all tissues other than the vasculature, therefore releasing basal resistance genes in these tissues from repression and allowing these cells to activate basal immunity in the case of spread of the infection. Long-distance signaling via the PEP pathway further contributes to this.

Finally, we looked for transcriptional regulators upstream of JA biosynthesis. For this, we chose the transcription factor gene *WRKY11* (pGG-PIP39). WRKY11 is a negative regulator of basal plant resistance, as well as a positive regulator of JA biosynthesis (Journot-Catalino *et al.*, 2006). Furthermore, *WRKY70*, a primary SA response gene, is under negative regulation by WRKY11 (Journot-Catalino *et al.*, 2006). Thus, WRKY11 may act as a positive regulator of JA biosynthesis, while also acting to restrict SA signaling to the HR zone, making it an intriguing marker in the context of this investigation. We found that under non-infected conditions, the *WRKY11* reporter is active throughout the plant in all tissues, as expected for a gene involved in negatively regulating basal resistance in sterile conditions (Fig. 6D). However, upon colonization of the vasculature by *F. oxysporum*, the pattern of *WRKY11* was confined to the vasculature only, with an apparent expression maximum in the cells close to the colonized cells (Fig. 6F). As these cells were not in immediate contact with the first colonized cells, this localized up-regulation indeed resembled the pattern of *AOS*. Intriguingly, expression of the reporter was now completely absent from the surrounding tissues, possibly to allow for the expression of basal resistance genes normally repressed by WRKY11 (Figs 6F, 7). Thus, transcriptional activation of JA biosynthesis and signaling overlaps with the activation of its regulator WRKY11.

## Discussion

The aim of this research was to develop the pGG-PIP vector set with 75 plant immune system promoters and demonstrate its usefulness by showing how these regulatory sequences can be used to image and monitor highly localized induction of a plant’s immune responses to infection by a pathogen.

Our pGG-PIP vector set, in combination with the original GreenGate kit from Lampropoulos *et al.* (2013) [and the updated GreenGate 2.0 and MultiGreenGate versions (Piepers *et al.*, 2023; Pennetti *et al.*, 2024)] is sufficient to build tools, such as the transcriptional reporter lines presented here, that can be probed for their responsiveness to a pathogen, elicitor, or other stimulant. Furthermore, owing to the universal compatibility of these entry vectors with all other GreenGate-based systems, this vector set can be used to screen for interactors of proteins in their native expression domain using the proximity ligation kit from Goslin *et al.* (2021, Preprint) or for tissue-specific gene knockouts by combining them with the CRISPR-TSKO kit from Decaestecker *et al.* (2019). The development and availability of these various toolboxes, all compatible with each other, demonstrate the usefulness of such modular cloning systems to enable researchers to quickly adopt new technologies, providing the flexibility and versatility to combine and recombine their existing vectors (i.e. modules) with new vectors from all other kits.

As a straightforward proof-of-principle, we used some of the pGG-PIPs to expand our knowledge of the roles of JA, ET, and SA in regulating plant defense against the fungal pathogen *F. oxysporum* to include spatially resolved transcriptional responses in roots. As previously described by Czymmek *et al.* (2007), we could confirm that *F. oxysporum* infects and colonizes *A. thaliana* almost exclusively via the root tip. We therefore reasoned that previous transcriptomic analyses using whole seedlings, or the entire root system, were insufficient to detect highly specific events occurring within the limited amount of infected tissue. This hypothesis was strengthened by our initial observation, namely that the infected tissue undergoes immediate cell death (Fig. 2A, B), thereby limiting the amount of infected tissue available for transcriptomic analyses. By employing a microscopy-based approach to live-image plant responses to infection in close to real time and at individual cell resolution, we were then able to show that JA, ET, and SA responses to colonization by *F. oxysporum* were targeted to clearly distinct groups of cells (Fig. 7).

We hypothesize that these distinct response zones represent a two-tiered defense mechanism of the plant. At the first tier, the plant initiates an HR via SA and ET in the cells immediately next to the colonization site, in a ‘burned-earth’ approach to defense, and to stop the pathogen from spreading. At the second tier, JA (and in part ET) initiates a second line of defense, in case the HR should fail to stop the invader. In this zone, which we designated the ‘defense response zone’ in our model, the plant may organize an active defense response to combat the invader (Fig. 7). Such ‘active’ defense could include ROS accumulation in the apoplast, an acidification of the apoplast via proton pumps, and the production of antimicrobial defense metabolites, including glucosinolates, camalexins, or coumarins (L. Wang *et al.*, 2022; Muñoz Hoyos and Stam, 2023).

Since our observations are based on the observation of transcriptional responses via reporter lines, this model is still hypothetical. However, we believe that the work reported here is an interesting and informative proof-of-principle to demonstrate how a microscopy-based approach to resolve the spatiality of the plant’s immune response can add understanding of the plant immune system. The fact that the highly targeted zones for JA and SA in response to colonization by *F. oxysporum* have so far been overlooked highlights the need for further high-resolution analysis to resolve the plant’s immune response to pathogenic infection on an individual cell level within intact tissue. By building on these initial results, using the pGG-PIP promoter collection that we have already donated to AddGene, and specifically in combination with the newly established spatial transcriptomic methodologies (Zhu *et al.*, 2023), it should be possible to further map the distinct spatial and temporal responses of other plant pathways to infection by *F. oxysporum* and any other pathogen, thereby creating a root atlas of the ‘plant immune system’.

## Supplementary data

The following supplementary data are available at *JXB* online.

Table 1. List of primers used to clone the plant immune system promoters.

Table 2. List of the 75 pGG-PIP promoter entry vectors in the set described in this paper.

erae516_suppl_Supplementary_Tables_S1-S2

## Data Availability

The 75 pGG-PIP GreenGate vectors have been deposited at AddGene (Deposit-ID: 82532, Catalog-#: 196739-196813)
