# Supplementary material for: Resolving spatially distinct phytohormone response zones in Arabidopsis thaliana roots colonized by Fusarium oxysporum
Source: J Exp Bot. 2025 Jan 17;76(7):2022–34. doi: 10.1093/jxb/erae516 (PMC12066115; doi:10.1093/jxb/erae516)
Supplement: erae516_suppl_Supplementary_Tables_S1-S2 [file erae516_suppl_supplementary_tables_s1-s2.pdf]

Supplementary Tables

Supplementary Table S1: List of primers used to clone the plant immune system promoters

The following primers were used to clone the 75 pGG-PIP putative regulatory sequences. They all contain the GreenGate overhang, allowing for recombination into the pGGA000 entry vector.

MAS-GG-ACS2p-F AACAGGTCTCAACCTCGGTCGATGTAAATGGATTAAATTTTATA
MAS-GG-ACS2p-R AACAGGTCTCATGTTGCTGTGTCAATTCTCACTTCTTTG
MAS-GG-ACS2pm-F AACAGGTCTCAGGTGTCCTCAAGGTTTCTGTTTCAAC
MAS-GG-ACS2pm-R AACAGGTCTCACACCAAATAAAGTTGATGTGGGGTC
MAS-GG-AOSp-F AACAGGTCTCAACCTACACTTAGACACCCCAATATTTTAGATTT
MAS-GG-AOSp-R AACAGGTCTCATGTTCTATTCGAAACAGTGGCGAGT
MAS-GG-AOSpm1-F AACAGGTCTCAGCCAATATTTTAGATTTTACTTTAAAGAAAT
MAS-GG-AOSpm1-R AACAGGTCTCATGGCGTCTCTAAGTGTTTTTTTTT
MAS-GG-AOSpm2-F AACAGGTCTCAGACACCTAAGTATTTTCTTTCCAACAA
MAS-GG-AOSpm2-R AACAGGTCTCATGTCCTTCTCTTTGAATAAACCTCA
MAS-GG-ARR5p-F AACAGGTCTCAACCTATATGATTTTTTCAAAAGAAAACACCATTTAGT
MAS-GG-ARR5p-R AACAGGTCTCATGTTATCAAGAAGAGTAGGATCGTGACTCGT
MAS-GG-ARR5pm-F AACAGGTCTCAGATTAGGATTATTCTTTATAGAATGTTTTGGTGC
MAS-GG-ARR5pm-R AACAGGTCTCAAATCGTCTCGGTTTTTACCTCTCAAATAGTAT
MAS-GG-ATG8Ap-F AACAGGTCTCAACCTTTAGCAGTGCTTAGTGAGCTTAAATTATAGTT
MAS-GG-ATG8Ap-R AACAGGTCTCATGTTAATTAATAAACTCGATCGTCTGCTAGATCG
MAS-GG-BAK1p-F AACAGGTCTCAACCTGTTTTTTGGAAACAGAGAGAAAACTCA
MAS-GG-BAK1p-R AACAGGTCTCATGTTTATCCTCAAGAGATTAAAAACAAACCCTA
MAS-GG-BIK1p-F AACAGGTCTCAACCTCGTTCCCAAATCTCGGTCAATTG
MAS-GG-BIK1p-R AACAGGTCTCATGTTCAAAGCTAAGAACAGATTCGTTTTCTTCTT
MAS-GG-BIK1pm-F AACAGGTCTCAGTACATTCATTTTGATTGGGATTTATCTTTTTA
MAS-GG-BIK1pm-R AACAGGTCTCAGTACAGACCAAAACAGTGGACTTGGG
MAS-GG-BPS1p-F AACAGGTCTCAACCTAAAAAGAGAGTCACTTGGGTTAAGTGATTT
MAS-GG-BPS1p-R AACAGGTCTCATGTTCTGATAAGTTTCAACTGAAAAAAACAAGAA
MAS-GG-CAD5p-F AACAGGTCTCAACCTGACTTGGGTTCAGTTAAAAATCTCA
MAS-GG-CAD5p-R AACAGGTCTCATGTTTTTGATGATTCTTTCTTCTTTCTTATC
MAS-GG-CERK1p-F AACAGGTCTCAACCTGTATGAAGAAGGGTAACAATTCAACTCTAA
MAS-GG-CERK1p-R AACAGGTCTCATGTTTGAAGCTTCCTTAGATTCCCCAGAGGAAGGGTGTCTGTT
MAS-GG-CIPP1p-F AACAGGTCTCAACCTCAAACCAGACATACTTGCAGCTTCTC
MAS-GG-CIPP1p-R AACAGGTCTCATGTTGAGAGTCAGATGTTCCACAAAAATCTACTC
MAS-GG-CIPP1pm-F AACAGGTCTCAGTTTGCAATCTGTACTAGAATAATATCGTGG
MAS-GG-CIPP1pm-R AACAGGTCTCAAAACGGATCTATATAACATATACGTATGCAATTAA
MAS-GG-CORK1p-F AACAGGTCTCAACCTGTACACTTATTAACATATATTTTTAATTTTGTG
MAS-GG-CORK1p-R AACAGGTCTCATGTTCGTCGACGACCAAAGATGTGAGA
MAS-GG-CORK1pm-F AACAGGTCTCAAGCCTGTTATTCTTAGTATTGCTTTCTATTTAAG
MAS-GG-CORK1pm-R AACAGGTCTCAGGCTTTAAACAATATAAAGCCCACAAG
MAS-GG-CPK29p-F AACAGGTCTCAACCTAATGAGGTAATGGAGGTTATCTTATCGA
MAS-GG-CPK29p-R AACAGGTCTCATGTTGTGAGCAAAGTAGATCGGTCTTCGA
MAS-GG-CPK29pm-F AACAGGTCTCAAGACACCCCCTCCAAGGGGCT
MAS-GG-CPK29pm-R AACAGGTCTCAGTCTCTAGGTTCTCCTATTCCTTTGCAA
MAS-GG-CPK5pro-F AACAGGTCTCAACCTACCATGTGACTACGACAACTACTGG
MAS-GG-CPK5pro-R AACAGGTCTCATGTTGAAACAATGGGAATTACCAAATCC
MAS-GG-CSLD2p-F AACAGGTCTCAACCTGACGACCAAGACTAGAGTTTTGGTTCG
MAS-GG-CSLD2p-R AACAGGTCTCATGTTAGTTAGGATCTAACTTGGCAGATCCCT
MAS-GG-DORN1p-F AACAGGTCTCAACCTGATGTAAAATTTGAAGCTTGAAGATGAAC
MAS-GG-DORN1p-R AACAGGTCTCATGTTGCAGATGATGAATCAGAGAGTCTGG
MAS-GG-EDS16p-F AACAGGTCTCAACCTGACTGCAGAGATCAATTTTCTTTTATTTTAT
MAS-GG-EDS16p-R AACAGGTCTCATGTTGCAGAAATTCGTAAAGTGTTTCTTGA
MAS-GG-EDS16pm1-F AACAGGTCTCACACCGCGTCCAACATTTTAAAACA

MAS-GG-EDS16pm1-R AACAGGTCTCAGGTGTCAGACTCTCAGCTGAACATAATT
MAS-GG-EDS16pm2-F AACAGGTCTCATCTGAAAGAGCCTAAGTGGGTTTCC
MAS-GG-EDS16pm2-R AACAGGTCTCACAGACCAGTTTTTATCATTTAAAAAATATTGTTA
MAS-GG-EDS1p-F AACAGGTCTCAACCTGTTTATCAGATTCCACGTACGATATGTTCTT
MAS-GG-EDS1p-R AACAGGTCTCATGTTGATCTATATCTATTCTCTTTTCTTTAGTGGACTTTC
MAS-GG-EDS1pm1-F AACAGGTCTCACGTGACCAAATCTGAAAACCCAAGT
MAS-GG-EDS1pm1-R AACAGGTCTCACACGTATAGAAGAAATCTACTACTTTAACTCTGTT
MAS-GG-EDS1pm2-F AACAGGTCTCACTCTCATGATGGGGTATTTTGGGTAAC
MAS-GG-EDS1pm2-R AACAGGTCTCAAGAGCATTTCAATGCAAAAATGGGT
MAS-GG-EFRp-F AACAGGTCTCAACCTATCTAGACGATTAAGTAATTGAGCATGTAAAAG
MAS-GG-EFRp-R AACAGGTCTCATGTTGTCGATTATAAAAAGATAAAAGAAAGGTTCTTCT
MAS-GG-ELI3p-F AACAGGTCTCAACCTTAAAGTCGATGTTCTATATGTATTCAAAATAAT
MAS-GG-ELI3p-R AACAGGTCTCATGTTATGGATAAATAATAAGCGAATGGGA
MAS-GG-ERF1p-F AACAGGTCTCAACCTCTCTCCCAATTGATATTTTTGTTATTTCT
MAS-GG-ERF1p-R AACAGGTCTCATGTTGTAGAAAAAAATACTCTGTTTCTTGACTACTCTGT
MAS-GG-EX1p-F AACAGGTCTCAACCTCCGCCGTCTTAAGTGGAATTTGG
MAS-GG-EX1p-R AACAGGTCTCATGTTCGCCGGAGAGATGTGAGAGCG
MAS-GG-FERp-F AACAGGTCTCAACCTAGAAAAGTTAAGAGTGGGAACTGGGA
MAS-GG-FERp-R AACAGGTCTCATGTTCGATCAAGAGCACTTCTCCGG
MAS-GG-FH16p-F AACAGGTCTCAACCTCTGACCATAGTGTGGTAACTCAGATATTTT
MAS-GG-FH16p-R AACAGGTCTCATGTTGGATCAGGAACCAAACAGATGATT
MAS-GG-FLS2p-F AACAGGTCTCAACCTTTTTTGTTAGAGAGATTTTTGTTGTTTTTGTT
MAS-GG-FLS2p-R AACAGGTCTCATGTTGGTTTAGACTTTAGAAGAGTTGAAATTGTGG
MAS-GG-FMO1p-F AACAGGTCTCAACCTCGGAAAAATCCTTCGTCAATGTGTG
MAS-GG-FMO1p-R AACAGGTCTCATGTTCTGAGAGAGGTTATGCTAGAGAGAAGAGAG
MAS-GG-FRK1p-F AACAGGTCTCAACCTAAATTAAACGCCTTTTTATCAACAAC
MAS-GG-FRK1p-R AACAGGTCTCATGTTACTTAATTGAGCTGCTTTCTCTGG
MAS-GG-FRK1pm-F AACAGGTCTCACGTCTCTTTACATTTGTGATGTGGT
MAS-GG-FRK1pm-R AACAGGTCTCAGACGAACACTGATATAAAAAATCTCACA
MAS-GG-GLR25p-F AACAGGTCTCAACCTGATAAGAAGTGATTCAGCTGGGGTTT
MAS-GG-GLR25p-R AACAGGTCTCATGTTATTGATAGCTCGCAAGCTCAAATCTG
MAS-GG-GLR25pm-F AACAGGTCTCAACCTCTCTCACCCTGGTCGCAAA
MAS-GG-GLR25pm-R AACAGGTCTCAAGGTTTTCTCTTTTCAACGTACAATATTTACATGA
MAS-GG-GLR27p-F AACAGGTCTCAACCTCACACACTGGTCACTTAATGGTTTATTTGA
MAS-GG-GLR27p-R AACAGGTCTCATGTTCCAGATTGAGGAACTTTATGTCATTCTTTAAC
MAS-GG-GLR27pm-F AACAGGTCTCAACTCAAAATGATGCGTATCATTAATTCTATAGTTT
MAS-GG-GLR27pm-R AACAGGTCTCAGAGTTTGAAATTTTTAACAAAAGTCTTAAGTTATATCAG
MAS-GG-GPAT5p-F AACAGGTCTCAACCTAAAAAGCGTTTTAATTAGAGAGATTTTTGC
MAS-GG-GPAT5p-R AACAGGTCTCATGTTCTTTTGTTTTTTGCTCGAATATTATTTT
MAS-GG-HPCA1p-F AACAGGTCTCAACCTAAACATAAGAGAAAACGCAAGTTGATGA
MAS-GG-HPCA1p-R AACAGGTCTCATGTTCTTCAAACCCAAAAAGAACCTCTTATCA
MAS-GG-HRMp-F AACAGGTCTCAACCTCTGAATATCTTCTTTTGGTTGCTCTGA
MAS-GG-HRMp-R AACAGGTCTCATGTTCGTAATATCTCTCTGTTTTTGCTCTGTTTT
MAS-GG-LECRKVI2p-F AACAGGTCTCAACCTACAAAGTCAATTACTTCGAGTTTTTTTCTG
MAS-GG-LECRKVI2p-R AACAGGTCTCATGTTGGGTGAGCGAAGTAAAGAAGGAGATA
MAS-GG-LYK5p-F AACAGGTCTCAACCTATTTTCTGTTAAGTTTGAACATTTGGTTGTAA
MAS-GG-LYK5p-R AACAGGTCTCATGTTTTGTGGTGTTCTGATCTGAAGAGG
MAS-GG-LYK5pm-F AACAGGTCTCACAACAGACCAAGACCATCTTTATGTCC
MAS-GG-LYK5pm-R AACAGGTCTCAGTTGGATCTCATGTGAAAGAGACACATT
MAS-GG-LYM1p-F AACAGGTCTCAACCTATATCATCGGTAAGTCACTAGACTATTGAACG
MAS-GG-LYM1p-R AACAGGTCTCATGTTTTGTGTTTAGGGTTTTACGAAATTCAA
MAS-GG-MIK2p-F AACAGGTCTCAACCTGTAAATAACGTTGAACTCGCGG
MAS-GG-MIK2p-R AACAGGTCTCATGTTACAGTTGCAGATTATCTCTCTACGGTC
MAS-GG-MLO6p-F AACAGGTCTCAACCTAAATATACATTTGGTTGACATGTTTCTCATT
MAS-GG-MLO6p-R AACAGGTCTCATGTTAGAACTCACAGAACAGTTCCAAGCAAA
MAS-GG-MPK3p-F AACAGGTCTCAACCTAAAAAAATTCTGATCGAAAATAGCTTAC

MAS-GG-MPK3p-R AACAGGTCTCATGTTCTCTCTCAATTGATCAAAGTCGA
MAS-GG-MPK4p-F AACAGGTCTCAACCTGACTTGTTTGTGAATATAGAGGAAACATGTAATTAT
MAS-GG-MPK4p-R AACAGGTCTCATGTTCGGAGCAAAATTCCTCACAACAACG
MAS-GG-MPK6p-F AACAGGTCTCAACCTAACACAAGAGAAGAGATTTATTGCTTC
MAS-GG-MPK6p-R AACAGGTCTCATGTTGACCGGTAAAGATGAAAGCTTTT
MAS-GG-MYB15p-F AACAGGTCTCAACCTGATGAATTTGAATAAACTAAACAAAATT
MAS-GG-MYB15p-R AACAGGTCTCATGTTCTCTTTGATTTGTGATTGCTGATAAA
MAS-MYB15m-F AGAGGACCATGGACACCTGAAGAAGATCAAATCTTT
MAS-GG-MYB72p-F AACAGGTCTCAACCTACACGATCTCTTTTGAGATTTAAGAAG
MAS-GG-MYB72p-R AACAGGTCTCATGTTCTTATTACACTACTTTCTTCTCTATAGCTACC
MAS-GG-MYB72pm1-F AACAGGTCTCACGTTTTAAAACTTTACCTTATGTCCAATCTCT
MAS-GG-MYB72pm1-R AACAGGTCTCAAACGTGACGTAGCATGTGTGGGTC
MAS-GG-MYB72pm2-F AACAGGTCTCAGCTCTCTCTCTACGAGTGAAGTGCCT
MAS-GG-MYB72pm2-R AACAGGTCTCAGAGCCAAAAGCATGGAACGTACG
MAS-GG-NET4Ap-F AACAGGTCTCAACCTTTAATCCTCTTCTCGTACATCACAT
MAS-GG-NET4Ap-R AACAGGTCTCATGTTGGCTGCAAAAATCAATGGACC
MAS-GG-PAD4p-F AACAGGTCTCAACCTAATTAGGGTTTTATCAGATTAAAGAGATTTACTGATT
MAS-GG-PAD4p-R AACAGGTCTCATGTTGATTGGATATCGAGTAGAGAGTTGCAGA
MAS-GG-PDF12p-F AACAGGTCTCAACCTTCTACCAAAAATCTTTGGTGCTTGATC
MAS-GG-PDF12p-R AACAGGTCTCATGTTGATGATTATTACTATTTTGTTTTCAATGTATAGA
MAS-GG-PEP1p-F AACAGGTCTCAACCTGAAGTCAAAAATTGAGTCGAAAAATC
MAS-GG-PEP1p-R AACAGGTCTCATGTTGAGATCTGATAAGACAGAGGAAAACTT
MAS-GG-PEP2p-F AACAGGTCTCAACCTTGAAGCTCTTGTGAATAGAGAAGAGA
MAS-GG-PEP2p-R AACAGGTCTCATGTTGAAATCCAATAGTTTGGTGAGTTATC
MAS-GG-PEP3p-F AACAGGTCTCAACCTGCACTTTAAGTTACATTGTTTAGTCTAATTATT
MAS-GG-PEP3p-R AACAGGTCTCATGTTCGTTGACTTCTTAATCTTTTTTTGGGAA
MAS-GG-PEPR1p-F AACAGGTCTCAACCTAGAGAAGGAAAACAACCATGTATTCCAG
MAS-GG-PEPR1p-R AACAGGTCTCATGTTCTGAGTTTAAAGATCGAGAAACATGCAG
MAS-GG-PEPR1pm-F AACAGGTCTCAGAAACCAAACATCTCGTCATAAAAAAC
MAS-GG-PEPR1pm-R AACAGGTCTCATTTCTCTGTATACCAACGATTGTGAGA
MAS-GG-PEPR2p-F AACAGGTCTCAACCTAGTTTGAGATGGAGTTGCATTGTG
MAS-GG-PEPR2p-R AACAGGTCTCATGTTGAGATTAGAGCTCAAGAGACTGAAATAT
MAS-GG-PER5p-F AACAGGTCTCAACCTCAGTGCGTAGTAGTGAGTTTTCTTCA
MAS-GG-PER5p-R AACAGGTCTCATGTTATTTGTAGATCTCACTTGGTATATATTTCGTAC
MAS-GG-PER5pm-F AACAGGTCTCAGACGAATATATATAATTAGCTACTAAATTAAATT
MAS-GG-PER5pm-R AACAGGTCTCACGTCTCAGAACGAGTGAATGATTC
MAS-GG-PLP1p-F AACAGGTCTCAACCTCTGATCATCTAGCCTCTTCCC
MAS-GG-PLP1p-R AACAGGTCTCATGTTAATAGTTGATCGATCTTCTTTTGAGTTAA
MAS-GG-PMR4p-F AACAGGTCTCAACCTGCTCGATGTCGATTTGAGACGTAGT
MAS-GG-PMR4p-R AACAGGTCTCATGTTAGTAGCATGTGGTAGATCTTAGAAATTTCTCG
MAS-GG-PR1pm-F AACAGGTCTCACTCCCTCCATATAAAAAAGTTTGATTTTATAG
MAS-GG-PR1pm-R AACAGGTCTCAGGAGAATCATTTTATAAGTTAAAACAAGCTTG
MAS-GG-PR1pro-F AACAGGTCTCAACCTATATATAACGATCATTGATTAGTATATATACATATTG
MAS-GG-PR1pro-R AACAGGTCTCATGTTTTCTAAGTTGATAATGGTTATTGTTGT
MAS-GG-RALF23p-F AACAGGTCTCAACCTGGTGATTCCGGTTTCCGACG
MAS-GG-RALF23p-R AACAGGTCTCATGTTTCTTCTGTACACTGTAGCTTTAGCTCTCTC
MAS-GG-RALF23pm-F AACAGGTCTCAGAGCACTCATAATTGTACAAAATAAAAGTAAATG
MAS-GG-RALF23pm-R AACAGGTCTCAGCTCTCCTTCCATGATTTGAGACTATTTC
MAS-GG-RBOHDp-F AACAGGTCTCAACCTGACTTGTTAAATTGCTCTCTTAGTCTTA
MAS-GG-RBOHDp-R AACAGGTCTCATGTTCGAATTCGAGAAACCAAAAAGATC
MAS-GG-RBHOFp-F AACAGGTCTCAACCTACCGGTTGAAAATAAGAGTGGTGGA
MAS-GG-RBOHFp-R AACAGGTCTCATGTTAGATCCAAAGTCGGAATTCAAAGAGTT
MAS-GG-RBOHFpm-F AACAGGTCTCATGCAGAAGATAGTGAAGATAGTTGCAGAA
MAS-GG-RBOHFpm-R AACAGGTCTCATGCAACTTTTATAGTTTTCGAACGAAAGTA
MAS-GG-RCD1p-F AACAGGTCTCAACCTGGAGGAGCAGATTGGACACCGT
MAS-GG-RCD1p-R AACAGGTCTCATGTTCTATATATTAACAATACTAAACCTATAACCTTGATAG

MAS-GG-RCD1pm-F AACAGGTCTCAATACGTCTCATATAGTTATGCTGATTCTTTCTTG
MAS-GG-RCD1pm-R AACAGGTCTCAGTATGATCCTGTAATATCATTCCTTCACAAAA
MAS-GG-RFO1p-F AACAGGTCTCAACCTATATTAACCATGCATGCAAACAAA
MAS-GG-RFO1p-R AACAGGTCTCATGTTTTTTTTTCTCTAATGACTTTTATGTATG
MAS-GG-RLP26p-F AACAGGTCTCAACCTGATTAAAGGATTGATCGGTAAACAAC
MAS-GG-RLP26p-R AACAGGTCTCATGTTGGTGTTTGTGATTGAACCAACAAGT
MAS-GG-RLP29p-F AACAGGTCTCAACCTCCAGCAAAAAGCTTCTTCTACTCAA
MAS-GG-RLP29p-R AACAGGTCTCATGTTAGGTTTTGGTGTAAGAGAGAGGAAAGA
MAS-GG-RPS4p-F AACAGGTCTCAACCTCGAGAACCTTGGCGAACTTGTCA
MAS-GG-RPS4p-R AACAGGTCTCATGTTGGCCCAAAAGCTTTTTCCCGGT
MAS-GG-SCOOP12p-F AACAGGTCTCAACCTAATAGGTTTCGAGTACTGTATTGATGTTTAACTG
MAS-GG-SCOOP12p-R AACAGGTCTCATGTTCTCGATCTTTATTTTTTTCTCGAGTTTAGA
MAS-GG-SOBIR1p-F AACAGGTCTCAACCTTTTCGATTTTTCTAATCTCACAGCTGTTC
MAS-GG-SOBIR1p-R AACAGGTCTCATGTTTAATTAGAGAAAGTTTCTTCTTGTGGATGTT
MAS-GG-SULTR41p-F AACAGGTCTCAACCTATGATCCATCACACGCCTGCCT
MAS-GG-SULTR41p-R AACAGGTCTCATGTTGATGGCTCTTGCGCACGCTTGG
MAS-GG-SULTR42p-F AACAGGTCTCAACCTGTAGCTTCCACGCCCTTGCCTAA
MAS-GG-SULTR42p-R AACAGGTCTCATGTTCGGAATTGGTGGGATAGAGAAGAAT
MAS-GG-TET8p-F AACAGGTCTCAACCTCGGATGTATCAAAGGTAAAAATATC
MAS-GG-TET8p-R AACAGGTCTCATGTTGGTTTAGATTCAGAGAGAAAGATTG
MAS-GG-TUB6p-F AACAGGTCTCAACCTATTTAGAGGGTGTTATTGGTTTGTG
MAS-GG-TUB6p-R AACAGGTCTCATGTTCTTCTATTTTATCTGAAATCAACATTACA
MAS-GG-TUB6pm1-F AACAGGTCTCATAACAAAAAGTTATGAATATTCACAGACATA
MAS-GG-TUB6pm1-R AACAGGTCTCAGTTATGGTTAACCGAGGATGAGC
MAS-GG-TUB6pm2-F AACAGGTCTCAGAGGCCATTTTTTTTTCCCGT
MAS-GG-TUB6pm2-R AACAGGTCTCACCTCATTGCGTATGACAATGCG
MAS-GG-VSP2p-F AACAGGTCTCAACCTTCTCTCTGGTTATATTTTGTTGCTGCTT
MAS-GG-VSP2p-R AACAGGTCTCATGTTGTTTTTTATGGTATGGTTTATTGTTTAGTTTGTG
MAS-GG-WAKL22p-F AACAGGTCTCAACCTATATTAACCATGCATGCAAACAAA
MAS-GG-WAKL22p-R AACAGGTCTCATGTTTTTTTTTCTCTAATGACTTTTATGTATG
MAS-GG-WRKY11p-F AACAGGTCTCAACCTTAGTTCCAAAACCGCATTGACAT
MAS-GG-WRKY11p-R AACAGGTCTCATGTTGATGATTTCTTGGTCTGAGGATTTT
MAS-GG-WRKY11pm-F AACAGGTCTCAGACGAAACTGTTGATTGCTTTATTCC
MAS-GG-WRKY11pm-R AACAGGTCTCACGTCTCCTCAAAGTTCGAGGTTACT
MAS-GG-WRKY17p-F AACAGGTCTCAACCTGTCTCGCAGAGGTTATTTATCTACTTGGTT
MAS-GG-WRKY17p-R AACAGGTCTCATGTTGATGAGAAACCAGAGGAGAAACTTGAAG
MAS-GG-WRKY17pm1-F AACAGGTCTCAGGTCAACGATTCCCATGTCGCTAA
MAS-GG-WRKY17pm1-R AACAGGTCTCAGACCTAACCGACTAATATATATGATTGTCGTG
MAS-GG-WRKY17pm2-F AACAGGTCTCAAAGCAGACCAAACTTTGATTACTTTATTCCATA
MAS-GG-WRKY17pm2-R AACAGGTCTCAGCTTGAGTTGTGAGATATGTAGGGTCTTCTT
MAS-GG-WRKY33p-F AACAGGTCTCAACCTCGCTGCTTTTTCGAGATAGATAG
MAS-GG-WRKY33p-R AACAGGTCTCATGTTACGAAAAATGGAAGTTTGTTTTATAA
MAS-GG-WRKY40p-F AACAGGTCTCAACCTTGTGTATAACTATTATGCAGCCTTTTTCAA
MAS-GG-WRKY40p-R AACAGGTCTCATGTTGTAAATATATGTAGGATGAATCTTCGATATGGGT
MAS-GG-WRKY40pm-F AACAGGTCTCATACAAGAATAGGTACAGTCCTGGTTTGTG
MAS-GG-WRKY40pm-R AACAGGTCTCATGTAATTGTGAATAATAAAATCTTAATTCAGAT
MAS-GG-WRKY53p-F AACAGGTCTCAACCTATCTTGTGAGCTGATTCAAAGATTTC
MAS-GG-WRKY53p-R AACAGGTCTCATGTTTTAGTATATGATTCCCAAAATAGATTTTTT
MAS-GG-WRKY70p-F AACAGGTCTCAACCTCATTGTAGATATGATATATGAAGCTTCCCC
MAS-GG-WRKY70p-R AACAGGTCTCATGTTGTTAGTTTTGAGGAAGTTTTTGGTGAG
MAS-GG-WRKY70pm-F AACAGGTCTCAGTATCTCGCATATTAACTTAGGCTAGAGAGC
MAS-GG-WRKY70pm-R AACAGGTCTCAATACTATGATAAACCAGTTGGTTCTGTAGCG
MAS-GG-XLG2p-F AACAGGTCTCAACCTGAGTGGAGGAGCATAGTGTGATTATTTAC
MAS-GG-XLG2p-R AACAGGTCTCATGTTCTTCTTACCCAATCAAGCACACATACAA

Supplementary Table S2: List of the 75 pGG-PIP promoter entry vectors in the set described in this paper.

A * next to the number in the Bases column indicates promoters for which we have cloned a described and tested DNA fragment, rather than the entire stretch to the neighboring gene.

GG-PIP plasmid	Gene	Bases	*Bsa*I site edits	Protein function	Full gene name	Gene code
pGG-PIP01	*PEP1*	1907		Activate defense genes (Yamada et al., 2016)	*PLANT ELICITOR PEPTIDE 1*	*AT5G64900*
pGG-PIP02	*PEP2*	769		Activate defense genes (Yamada et al., 2016)	*PLANT ELICITOR PEPTIDE 2*	*AT5G64890*
pGG-PIP03	*PEP3*	1694		Activate defense genes (Yamada et al., 2016)	*PLANT ELICITOR PEPTIDE 3*	*AT5G64905*
pGG-PIP04	*RALF23*	2027	615 C->G	Phytosulfokine sensed by FER (Stegmann et al., 2017)	*ARABIDOPSIS RAPID ALKALINIZATION FACTOR 23*	*AT3G16570*
pGG-PIP05	*SCOOP12*	2900		Phytosulfokine sensed by MIK2/BAK1 (Rhodes et al., 2021; Hou et al., 2021)	*PRECURSOR OF SERINE-RICH ENDOGENOUS PEPTIDE 12*	*AT5G44585*
pGG-PIP06	*BAK1*	1731		Co-receptor for several defense & development pathways (Greenwood and Williams, 2022)	*BRI1-ASSOCIATED RECEPTOR KINASE*	*AT4G33430*
pGG-PIP07	*CERK1*	493*		Chitin-receptor (Cao et al., 2014)	*CHITIN ELICITOR RECEPTOR KINASE 1*	*AT3G21630*
pGG-PIP08	*CIPP1*	3003		CERK1 co-receptor (Liu et al., 2018)	*CERK-1 INTERACTING PROTEIN PHOSPHATASE 1*	*AT1G34750*
pGG-PIP09	*CORK1*	3376		DAMP-receptor (Tseng et al., 2022)	*CELLOOLIGOMER RECEPTOR KINASE 1*	*AT1G56145*
pGG-PIP10	*EFR*	2376		Receptor for EF-Tu (Couto and Zipfel, 2016)	*EF-TU RECEPTOR*	*AT5G20480*
pGG-PIP11	*FER*	1243		Co-receptor for several defense & development pathways (Duan et al., 2022)	*FERONIA*	*AT3G51550*
pGG-PIP12	*FLS2*	2913		Receptor for bacterial flagellin (Couto and Zipfel, 2016)	*FLAGELLIN-SENSITIVE 2*	*AT5G46330*
pGG-PIP13	*LYK5*	1560	346 G->C	CERK1 co-receptor (Cao et al., 2014)	*LYSM-CONTAINING RECEPTOR-LIKE KINASE 5*	*AT2G33580*
pGG-PIP14	*LYM1*	991		Fungal MAMP receptor (Zipfel and Oldroyd, 2017)	*LYSM DOMAIN GPI-ANCHORED PROTEIN 1 PRECURSOR*	*AT1G21880*
pGG-PIP15	*MIK2*	2513		SCOOP peptide receptor (Rhodes et al., 2021; Hou et al., 2021)	*MALE DISCOVERER 1-INTERACTING RECEPTOR-LIKE KINASE 2*	*AT4G08850*
pGG-PIP16	*PEPR1*	931	180 G->A	Receptor for PEP1-6 (Yamada et al., 2016)	*PEP1 RECEPTOR 1*	*AT1G73080*
pGG-PIP17	*PEPR2*	1676		Receptor for PEP1 & 2 (Yamada et al., 2016)	*PEP1 RECEPTOR 2*	*AT1G17750*
pGG-PIP18	*RFO1*	850		Cell wall-associated kinase (Huerta et al., 2023)	*RESISTANCE TO FUSARIUM OXYSPORUM 1*	*AT1G79670*
pGG-PIP19	*RLP26*	822		Co-receptor for PRR receptors (Wu et al., 2016)	*RECEPTOR LIKE PROTEIN 26*	*AT2G33050*
pGG-PIP20	*RLP29*	2790		Co-receptor for PRR receptors (Wu et al., 2016)	*RECEPTOR LIKE PROTEIN 29*	*AT2G42800*
pGG-PIP21	*SOBIR1*	1163		Co-receptor for several defense pathways (Cho et al., 2023)	*SUPPRESSOR OF BIR 1 / EVERSHED*	*AT2G31880*
pGG-PIP22	*BIK1*	2668	1878 C->G	Defense signaling (Gonçalves Dias et al., 2022)	*BOTRYTIS-INDUCED KINASE1*	*AT2G39660*
pGG-PIP23	*XLG2*	1256		G protein involved in immunity (Petutschnig et al., 2022)	*EXTRA-LARGE GTP-BINDING PROTEIN 2*	*AT4G34390*
pGG-PIP24	*MPK3*	654		Activates immune response (Tsuda and Somssich, 2015)	*MITOGEN-ACTIVATED PROTEIN KINASE 3*	*AT3G45640*
pGG-PIP25	*MPK4*	1025		Activates immune response (Tsuda and Somssich, 2015)	*MITOGEN-ACTIVATED PROTEIN KINASE 4*	*AT4G01370*
pGG-PIP26	*MPK6*	811		Activates immune response (Tsuda and Somssich, 2015)	*MITOGEN-ACTIVATED PROTEIN KINASE 6*	*AT2G43790*
pGG-PIP27	*CPK5*	1983		Involved in calcium-dependent stress responses (Gao and He, 2013)	*CALMODULIN-DOMAIN PROTEIN KINASE 5*	*AT4G35310*

pGG-PIP28	*CPK29*	826	220 G->C	Involved in calcium-dependent stress responses (Patil and Senthil-Kumar, 2020)	*CALCIUM-DEPENDENT PROTEIN KINASE 29*	*AT1G76040*
pGG-PIP29	*GLR2.5*	2488	1488, 1489 G->C	Involved in defense-development balancing (Birkenbihl et al., 2017a)	*GLUTAMATE RECEPTOR 2.5*	*AT5G11210*
pGG-PIP30	*GLR2.7*	4000*		Involved in defense-development balancing (Birkenbihl et al., 2017a)	*GLUTAMATE RECEPTOR 2.7*	*AT2G29120*
pGG-PIP31	*RBOHD*	2309		Produces ROS-burst (Kadota et al., 2014)	*RESPIRATORY BURST OXIDASE HOMOLOGUE D*	*AT5G47910*
pGG-PIP32	*RBOHF*	4119		Produces ROS-burst (Morales et al., 2016)	*RESPIRATORY BURST OXIDASE HOMOLOG F*	*AT1G64060*
pGG-PIP33	*EX1*	989		Produces ROS from chloroplasts (Dogra et al., 2022)	*EXECUTER 1*	*AT4G33630*
pGG-PIP34	*HPCA1*	2944		ROS-sensor (Sun and Zhang, 2021)	*HP-INDUCED Ca2+ INCREASES 1*	*AT5G49760*
pGG-PIP35	*RCD1*	3137	248 G->C	Modulator of root-to-shoot ROS-signaling (Jin et al., 2022)	*RADICAL-INDUCED CELL DEATH1*	*AT1G32230*
pGG-PIP36	*DORN1*	2325		eATP DAMP-receptor (Sun and Zhang, 2021)	*DOES NOT RESPOND TO NUCLEOTIDES 1*	*AT5G60300*
pGG-PIP37	*LECRK-VI.2*	1183		NAD(P)-receptor (Sun and Zhang, 2021)	*L-TYPE LECTIN RECEPTOR KINASE-VI.2*	*AT5G01540*
pGG-PIP38	*BPS1*	2142		Regulator of root-to-shoot communication (Lee et al., 2016)	*BYPASS 1*	*AT1G01550*
pGG-PIP39	*WRKY11*	2026	240 C->G	Regulates defense gene expression (Journot-Catalino et al., 2006)	*WRKY DNA-BINDING PROTEIN 11*	*AT4G31550*
pGG-PIP40	*WRKY17*	4565	241 G->C	Regulates defense gene expression (Birkenbihl et al., 2018)	*WRKY DNA-BINDING PROTEIN 17*	*AT2G24570*
pGG-PIP41	*WRKY33*	1665		Regulates defense gene expression (Birkenbihl et al., 2018)	*WRKY DNA-BINDING PROTEIN 33*	*AT4G23810*
pGG-PIP42	*WRKY40*	4158		Regulates defense gene expression (Birkenbihl et al., 2018)	*WRKY DNA-BINDING PROTEIN 40*	*AT1G80840*
pGG-PIP43	*WRKY53*	2515		Regulates defense gene expression (Birkenbihl et al., 2017b)	*WRKY DNA-BINDING PROTEIN 53*	*AT2G38470*
pGG-PIP44	*WRKY70*	4099		Regulates defense gene expression (Journot-Catalino et al., 2006)	*WRKY DNA-BINDING PROTEIN 70*	*AT3G56400*
pGG-PIP45	*ELI-3*	524		Elicitor-response gene (Tanaka et al., 2018)	*CINNAMYL-ALCOHOL DEHYDROGENASE 7 / ELICITOR-ACTIVATED GENE3*	*AT4G37980*
pGG-PIP46	*FRK1*	1271	493 G->C	Defense gene (Birkenbihl et al., 2017b)	*FLG22-INDUCED RECEPTOR-LIKE KINASE 1*	*AT2G19190*
pGG-PIP47	*MLO6*	3042		Defense gene (Acevedo-Garcia et al., 2017)	*MILDEW RESISTANCE LOCUS O 6*	*AT1G61560*
pGG-PIP48	*PER5*	2007	558 C->G	Marker for activated immune signaling (Chuberre et al., 2018)	*PEROXIDASE 5*	*AT1G14550*
pGG-PIP49	*PLP1*	2490		Pathogen-induced (Yang et al., 2007)	*PATATIN-LIKE PROTEIN 1*	*AT4G37070*
pGG-PIP50	*ACS2*	2914	150 C->G	ET biosynthesis (Wang et al., 2022b)	*1-AMINO-CYCLOPROPANE-1-CARBOXYLATE SYNTHASE 2*	*AT1G01480*
pGG-PIP51	*AOS*	2093	2088 G->C ; 1434 G->C	JA biosynthesis (Yang et al., 2019b)	*ALLENE OXIDE SYNTHASE*	*AT5G42650*
pGG-PIP52	*ARR5*	2229	1591 C->G	Cytokinin signaling (Lee et al., 2016)	*ARABIDOPSIS RESPONSE REGULATOR 5*	*AT3G48100*
pGG-PIP53	*EDS16*	2975	1579 G->C ;	SA biosynthesis (Genger et al., 2008)	*ENHANCED DISEASE SUSCEPTIBILITY TO ERYSIPHE ORONTII 16*	*AT1G74710*

			1049 C->G			
pGG-PIP54	*ERF1*	2682		ET & JA response regulator (Wang et al., 2022b)	*ETHYLENE RESPONSE FACTOR 1*	*AT3G23240*
pGG-PIP55	*PDF1.2*	1540		ET & JA-induced defense gene (Yang et al., 2019b)	*PLANT DEFENSIN 1.2*	*AT5G44420*
pGG-PIP56	*PR1*	2343		SA-induced defense gene (Yang et al., 2019b)	*PATHOGENESIS-RELATED GENE 1*	*AT2G14610*
pGG-PIP57	*VSP2*	1471		JA-induced defense gene (Yang et al., 2019b)	*VEGETATIVE STORAGE PROTEIN 2*	*AT5G24770*
pGG-PIP58	*FMO1*	1726*		Systemic acquired resistance marker (Joglekar et al., 2018)	*FLAVIN-DEPENDENT MONOOXYGENASE 1*	*AT1G19250*
pGG-PIP59	*MYB72*	4619		Involved in induced systemic resistance	*ARABIDOPSIS THALIANA MYB DOMAIN PROTEIN 72*	*AT1G56160*
pGG-PIP60	*CSLD2*	1963		Marker for cell death, possibly via ET-signaling (Salguero-Linares et al., 2022)	*CELLULOSE-SYNTHASE LIKE D2*	*AT5G16910*
pGG-PIP61	*HRM1*	2238		Marker for cell death (Salguero-Linares et al., 2022)	*HYPERSENSITIVE RESPONSE MARKER 1*	*AT5G17760*
pGG-PIP62	*EDS1*	1419	151 G->C ; 572 A->T	Involved in ETI-signaling (Jia et al., 2022)	*ENHANCED DISEASE SUSCEPTIBILITY 1*	*AT3G48090*
pGG-PIP63	*PAD4*	1596		Involved in ETI-signaling (Jia et al., 2022)	*PHYTOALEXIN DEFICIENT 4*	*AT3G52430*
pGG-PIP64	*RPS4*	511*		NLR effector receptor (Jia et al., 2022)	*RESISTANT TO P. SYRINGAE 4*	*AT5G45250*
pGG-PIP65	*ATG8A*	1408		Autophagy marker (Yang et al., 2019a)	*AUTOPHAGY-RELATED 8A*	*AT4G21980*
pGG-PIP66	*TET8*	2068		Targets vesicular transport to infection sites (Cai et al., 2018)	*TETRASPANIN8*	*AT2G23810*
pGG-PIP67	*SULTR4;1*	2087		Sulfate transporter (Wang et al., 2022a)	*SULFATE TRANSPORTER 4.1*	*AT5G13550*
pGG-PIP68	*SULTR4;2*	712		Sulfate transporter (Wang et al., 2022a)	*SULFATE TRANSPORTER 4.2*	*AT3G12520*
pGG-PIP69	*CAD5*	1134		Lignin biosynthesis (Kim et al., 2020)	*CINNAMYL ALCOHOL DEHYDROGENASE 5*	*AT4G34230*
pGG-PIP70	*GPAT5*	1587		Suberin biosynthesis (Andersen et al., 2015)	*GLYCEROL-3-PHOSPHATE SN-2-ACYLTRANSFERASE 5*	*AT3G11430*
pGG-PIP71	*MYB15*	2050*		Lignin biosynthesis in response to pathogens (Kim et al., 2020)	*ARABIDOPSIS THALIANA MYB DOMAIN PROTEIN 15*	*AT3G23250*
pGG-PIP72	*PMR4*	1954		PAMP-induced defense, callose-deposition (Blümke et al., 2013)	*POWDERY MILDEW RESISTANT 4*	*AT4G03550*
pGG-PIP73	*FH16*	1158		Involved in microtubule/actin-network reorganization (Wang et al., 2013)	*FORMIN HOMOLOG 16*	*AT5G07770*
pGG-PIP74	*NET4A*	1718		Modulates the vacuole (Kaiser et al., 2019)	*NETWORKED 4A*	*AT5G58320*
pGG-PIP75	*TUB6*	3349	992 A->G	Component of microtubules (Liu et al., 2019)	*BETA-6 TUBULIN*	*AT5G12250*

Suppl. References

Acevedo-Garcia, J. et al. (2017). The powdery mildew-resistant Arabidopsis mlo2 mlo6 mlo12 triple mutant displays altered infection phenotypes with diverse types of phytopathogens. Sci. Rep. **7**: 9319.

Andersen, T.G., Barberon, M., and Geldner, N. (2015). Suberization-the second life of an endodermal cell. Curr. Opin. Plant Biol. **28**: 9–15.

Birkenbihl, R.P., Kracher, B., Roccaro, M., and Somssich, I.E. (2017a). Induced genome-wide binding of three Arabidopsis WRKY transcription factors during early MAMP-triggered immunity. Plant Cell **29**: 20–38.

Birkenbihl, R.P., Kracher, B., Ross, A., Kramer, K., Finkemeier, I., and Somssich, I.E. (2018). Principles and characteristics of the Arabidopsis WRKY regulatory network during early MAMP-triggered immunity. Plant J. **96**: 487–502.

Birkenbihl, R.P., Liu, S., and Somssich, I.E. (2017b). Transcriptional events defining plant immune responses. Curr. Opin. Plant Biol. **38**: 1–9.

Blümke, A., Somerville, S.C., and Voigt, C.A. (2013). Transient expression of the Arabidopsis thaliana callose synthase PMR4 increases penetration resistance to powdery mildew in barley. Adv. Biosci. Biotechnol. **04**: 810–813.

Cai, Q., Qiao, L., Wang, M., He, B., Lin, F., Palmquist, J., Huang, S.-D., and Jin, H. (2018). Plants send small RNAs in extracellular vesicles to fungal pathogen to silence virulence genes. Science (80-.). **360**: 1126–1129.

Cao, Y., Liang, Y., Tanaka, K., Nguyen, C.T., Jedrzejczak, R.P., Joachimiak, A., and Stacey, G. (2014). The kinase LYK5 is a major chitin receptor in Arabidopsis and forms a chitin-induced complex with related kinase CERK1. Elife **3**: 1–19.

Cho, H., Lee, J., and Oh, E. (2023). Leucine-rich repeat receptor-like proteins in plants: Structure, function, and signaling. J. Plant Biol. **66**: 99–107.

Chuberre, C., Plancot, B., Driouich, A., Moore, J.P., Bardor, M., Gügi, B., and Vicré, M. (2018). Plant immunity is compartmentalized and specialized in roots. Front. Plant Sci. **9**: 1–13.

Couto, D. and Zipfel, C. (2016). Regulation of pattern recognition receptor signalling in plants. Nat. Rev. Immunol. **16**: 537–552.

Dogra, V., Singh, R.M., Li, M., Li, M., Singh, S., and Kim, C. (2022). EXECUTER2 modulates the EXECUTER1 signalosome through its singlet oxygen-dependent oxidation. Mol. Plant **15**: 438–453.

Duan, Z., Liu, W., Li, K., Duan, W., Zhu, S., Xing, J., Chen, T., and Luo, X. (2022). Regulation of immune complex formation and signalling by FERONIA, a busy goddess in plant–microbe interactions. Mol. Plant Pathol. **23**: 1695–1700.

Gao, X. and He, P. (2013). Nuclear dynamics of Arabidopsis calcium-dependent protein kinases in effector-triggered immunity. Plant Signal. Behav. **8**: e23868.

Genger, R.K., Jurkowski, G.I., McDowell, J.M., Lu, H., Ho, W.J., Greenberg, J.T., and Bent, A.F. (2008). Signaling pathways that regulate the enhanced disease resistance of Arabidopsis "defense, no death" mutants. Mol. Plant-Microbe Interact. **21**: 1285–1296.

Gonçalves Dias, M., Soleimani, F., and Monaghan, J. (2022). Activation and turnover of the plant immune signaling kinase BIK1: a fine balance. Essays Biochem.: 1–12.

Greenwood, J.R. and Williams, S.J. (2022). Guarding the central regulator of extracellular perception in plants – A job for two. Cell Host Microbe **30**: 1657–1659.

Hou, S. et al. (2021). The Arabidopsis MIK2 receptor elicits immunity by sensing a conserved signature from phytocytokines and microbes. Nat. Commun. **12**: 5494.

Huerta, A.I. et al. (2023). The WAK-like protein RFO1 acts as a sensor of the pectin methylation status in Arabidopsis cell walls to modulate root growth and defense. Mol. Plant **2**: 33–47.

Jia, A. et al. (2022). TIR-catalyzed ADP-ribosylation reactions produce signaling molecules for plant immunity. Science (80-.). **377**: eabq8180.

Jin, T., Wu, H., Deng, Z., Cai, T., Li, J., Liu, Z., Waterhouse, P.M., White, R.G., and Liang, D. (2022). Control of root-to-shoot long-distance flow by a key ROS-regulating factor in Arabidopsis. Plant. Cell Environ. **45**: 2476–2491.

Joglekar, S., Suliman, M., Bartsch, M., Halder, V., Maintz, J., Bautor, J., Zeier, J., Parker, J.E., and Kombrink, E. (2018). Chemical activation of EDS1/PAD4 signaling leading to pathogen resistance in Arabidopsis. Plant Cell Physiol. **59**: 1592–1607.

Journot-Catalino, N., Somssich, I.E., Roby, D., and Kroj, T. (2006). The transcription factors WRKY11 and WRKY17 act as negative regulators of basal resistance in Arabidopsis thaliana. Plant Cell **18**: 3289–3302.

Kadota, Y., Sklenar, J., Derbyshire, P., Stransfeld, L., Asai, S., Ntoukakis, V., Jones, J.D.G., Shirasu, K., Menke, F.L.H., Jones, A.M.E., and Zipfel, C. (2014). Direct regulation of the NADPH oxidase RBOHD by the PRR-associated kinase BIK1 during plant immunity. Mol. Cell **54**: 43–55.

Kaiser, S., Eisa, A., Kleine-Vehn, J., and Scheuring, D. (2019). NET4 modulates the compactness of vacuoles in Arabidopsis thaliana. Int. J. Mol. Sci. **20**: 4752.

Kim, S.H., Lam, P.Y., Lee, M.-H., Jeon, H.S., Tobimatsu, Y., and Park, O.K. (2020). The Arabidopsis R2R3 MYB transcription factor MYB15 is a key regulator of lignin biosynthesis in effector-triggered immunity. Front. Plant Sci. **11**: 1–10.

Lee, D.-K., Parrott, D.L., Adhikari, E., Fraser, N., and Sieburth, L.E. (2016). The mobile bypass signal arrests shoot growth by disrupting shoot apical meristem maintenance, cytokinin signaling, and WUS transcription factor expression. Plant Physiol. **171**: 2178–2190.

Liu, J., Liu, B., Chen, S., Gong, B.-Q., Chen, L., Zhou, Q., Xiong, F., Wang, M., Feng, D., Li, J.-F., Wang, H.-B., and Wang, J. (2018). A tyrosine phosphorylation cycle regulates fungal activation of a plant receptor Ser/Thr kinase. Cell Host Microbe **23**: 241-253.e6.

Liu, W., Wang, C., Wang, G., Ma, Y., Tian, J., Yu, Y., Dong, L., and Kong, Z. (2019). Towards a better recording of microtubule cytoskeletal spatial organization and dynamics in plant cells. J. Integr. Plant Biol. **61**: 388–393.

Morales, J., Kadota, Y., Zipfel, C., Molina, A., and Torres, M.-A. (2016). The Arabidopsis NADPH oxidases RbohD and RbohF display differential expression patterns and contributions during plant immunity. J. Exp. Bot. **67**: 1663–1676.

Patil, M. and Senthil-Kumar, M. (2020). Role of plant kinases in combined stress. In Protein Kinases and Stress Signaling in Plants (Wiley), pp. 445–458.

Petutschnig, E.K., Anders, J., Stolze, M., Meusel, C., Hacke, R., Much, L., Schwier, M., Gippert, A.-L., Kroll, S., Fasshauer, P., Wiermer, M., and Lipka, V. (2022). EXTRA LARGE G-PROTEIN2 mediates cell death and hyperimmunity in the chitin elicitor receptor kinase 1-4 mutant. Plant Physiol. **189**: 2413–2431.

Rhodes, J., Yang, H., Moussu, S., Boutrot, F., Santiago, J., and Zipfel, C. (2021). Perception of a divergent family of phytocytokines by the Arabidopsis receptor kinase MIK2. Nat. Commun. **12**: 705.

Salguero-Linares, J., Serrano, I., Ruiz-Solani, N., Salas-Gómez, M., Phukan, U.J., González, V.M., Bernardo-Faura, M., Valls, M., Rengel, D., and Coll, N.S. (2022). Robust transcriptional indicators of immune cell death revealed by spatiotemporal transcriptome analyses. Mol. Plant **15**: 1059–1075.

Stegmann, M., Monaghan, J., Smakowska-Luzan, E., Rovenich, H., Lehner, A., Holton, N.J., Belkhadir, Y., and Zipfel, C. (2017). The receptor kinase FER is a RALF-regulated scaffold controlling plant immune signaling. Science (80-.). **355**: 287–289.

Sun, T. and Zhang, Y. (2021). Short- and long-distance signaling in plant defense. Plant J. **105**: 505–517.

Tanaka, T. et al. (2018). Identification of a hexenal reductase that modulates the composition of green leaf volatiles. Plant Physiol. **178**: 552–564.

Tseng, Y.-H., Scholz, S.S., Fliegmann, J., Krüger, T., Gandhi, A., Furch, A.C.U., Kniemeyer, O., Brakhage, A.A., and Oelmüller, R. (2022). CORK1, A LRR-Malectin Receptor Kinase, is required for cellooligomer-induced responses in Arabidopsis thaliana. Cells **11**: 2960.

Tsuda, K. and Somssich, I.E. (2015). Transcriptional networks in plant immunity. New Phytol. **206**: 932–47.

Wang, J., Zhang, Y., Wu, J., Meng, L., and Ren, H. (2013). AtFH16, an Arabidopsis type II formin, binds and bundles both microfilaments and microtubules, and preferentially binds to microtubules. J. Integr. Plant Biol. **55**: 1002–1015.

Wang, W., Liu, J., Mishra, B., Mukhtar, M.S., and McDowell, J.M. (2022a). Sparking a sulfur war between plants and pathogens. Trends Plant Sci. **27**: 1253–1265.

Wang, X., Meng, H., Tang, Y., Zhang, Y., He, Y., Zhou, J., and Meng, X. (2022b). Phosphorylation of an ethylene response factor by MPK3/MPK6 mediates negative

feedback regulation of pathogen-induced ethylene biosynthesis in Arabidopsis. J. Genet. Genomics **49**: 810–822.

Wu, J., Liu, Z., Zhang, Z., Lv, Y., Yang, N., Zhang, G., Wu, M., Lv, S., Pan, L., Joosten, M.H.A.J., and Wang, G. (2016). Transcriptional regulation of receptor-like protein genes by environmental stresses and hormones and their overexpression activities in Arabidopsis thaliana. J. Exp. Bot. **67**: 3339–3351.

Yamada, K., Yamashita-Yamada, M., Hirase, T., Fujiwara, T., Tsuda, K., Hiruma, K., and Saijo, Y. (2016). Danger peptide receptor signaling in plants ensures basal immunity upon pathogen-induced depletion of BAK1. EMBO J. **35**: 46–61.

Yang, F., Kimberlin, A.N., Elowsky, C.G., Liu, Y., Gonzalez-Solis, A., Cahoon, E.B., and Alfano, J.R. (2019a). A plant immune receptor degraded by selective autophagy. Mol. Plant **12**: 113–123.

Yang, J., Duan, G., Li, C., Liu, L., Han, G., Zhang, Y., and Wang, C. (2019b). The crosstalks between jasmonic acid and other plant hormone signaling highlight the involvement of jasmonic acid as a core component in plant response to biotic and abiotic stresses. Front. Plant Sci. **10**: 1–12.

Yang, W., Devaiah, S.P., Pan, X., Isaac, G., Welti, R., and Wang, X. (2007). AtPLAI is an acyl hydrolase involved in basal jasmonic acid production and Arabidopsis resistance to Botrytis cinerea. J. Biol. Chem. **282**: 18116–18128.

Zipfel, C. and Oldroyd, G.E.D. (2017). Plant signalling in symbiosis and immunity. Nature **543**: 328–336.
